# Validation of the Copenhagen Psychosocial Questionnaire Version III and Establishment of Benchmarks for Psychosocial Risk Management in Sweden

**DOI:** 10.3390/ijerph17093179

**Published:** 2020-05-02

**Authors:** Hanne Berthelsen, Hugo Westerlund, Gunnar Bergström, Hermann Burr

**Affiliations:** 1 Centre for Work Life and Evaluation Studies (CTA) & the Faculty of Odontology, Malmö University, 205 06 Malmö, Sweden; 2 Department of Psychology, Stress Research Institute, Stockholm University, 106 91 Stockholm, Sweden; hugo.westerlund@su.se; 3 Department of Occupational Health Sciences and Psychology, Centre for Musculoskeletal Research, University of Gävle, 801 76 Gävle, Sweden; gunnar.bergstrom@hig.se; 4 Institute of Environmental Medicine, Unit of Intervention and Implementation Research for Worker Health, Karolinska Institute, 171 77 Stockholm, Sweden; 5 Division 3 Work and Health, Federal Institute of Occupational Safety and Health (BAuA), 10317 Berlin, Germany; burr.hermann@baua.bund.de

**Keywords:** psychosocial risk assessment, psychosocial risk management, benchmark, organizational and social work environment, psychometric evaluation, occupational health

## Abstract

This study presents the Swedish standard version of the Copenhagen Psychosocial Questionnaire, COPSOQ III, and investigates its reliability and validity at individual and workplace levels with the aim of establishing benchmarks for the psychosocial work environment. Cross-sectional data from (1) a random sample of employees in Sweden aged 25–65 years (N = 2847) and (2) a convenience sample of non-managerial employees at 51 workplaces (N = 1818) were analysed. Internal consistency reliability was evaluated as well as the effects of sex, work sector and blue/white-collar work. Population benchmarks and mean scores for major occupational groups were computed based on weighted data. ICC(1) and ICC(2) estimates were computed to evaluate aggregation to the workplace level and Pearson inter-correlations to evaluate construct validity at individual and aggregated levels. The reliability and scale characteristics were satisfactory, with few exceptions, at both individual and workplace levels. The strength and direction of correlations supported the construct validity of the dimensions and the amount of variance explained by workplace justified aggregation to the workplace level. The present study thus supports the use of COPSOQ III for measurement at the workplace level and presents benchmarks for risk management as well as for research purposes.

## 1. Introduction

Measuring the psychosocial work environment in a valid and reliable way is increasingly seen as a necessary part of systematic occupational safety and health management [1,2,3,4]. A widely used research-based non-commercial tool for psychosocial workplace surveys is the Copenhagen Psychosocial Questionnaire (COPSOQ). Originally developed in 2000 for use in research and at workplaces in Denmark, it has today been validated in 18 countries, and results from research from even more language versions have been reported in hundreds of peer-reviewed articles [5]. COPSOQ is intended for both workplace measurement, usually comparing work groups, departments or companies, and for research, e.g., investigating effects of work environment on health or labour market attainment. The International COPSOQ Network recently released a revised third version, COPSOQ III [5], which is an update of the two previous versions of the instrument [6,7]. The changes are primarily based on experiences from practical use of previous versions for workplace assessments and research but have also taken labour market changes and theoretical developments into consideration [5]. Importantly, the new version is designed to allow flexible adaptation to national and industry-specific contexts without compromising the potential for international comparisons and for comparisons over time. Items labelled as “core”, “middle” and “long” compose the international COPSOQ III structure. While inclusion of core items is mandatory for national versions, it is important to underline that they do not constitute a short version of the instrument. National versions can be established by the national COPSOQ teams of each country based on all “core” items supplemented with enough items labelled as “middle” or “long” to form a reliable and relevant measurement in the given context. Therefore, all future national versions will include the same mandatory core items, while the total number of items in scales and number of scales are allowed to differ [5].

The new Swedish standard version of COPSOQ III is based on preceding development, adaptation and testing of COPSOQ II for use at workplaces and research in the Swedish context [8,9,10] also taking the new COPSOQ III into account [5]. Several studies have corroborated different aspects of reliability and validity of the Swedish version of COPSOQ II. An iterative process including translation-back-translation procedures and cognitive interviewing methods supported the face and content validity, as well as the cross-cultural equivalency of COPSOQ II and COPSOQ III test items [8,9,10]. The nomological validity has been corroborated by operationalization of an extended JD-R model by the instrument with aspects of workability as outcome [11] as well as need for recovery [12] and also in relation to the newly introduced dimensions in the COPSOQ III of Work Engagement, Quality of Work [13] and Cyber Bullying [14]. Studies across different occupations have corroborated the internal consistency reliability and construct validity of the scales [11,12,13,15,16,17,18,19]. The ability to distinguish different groups (organizations with similar missions, work teams or occupational groups) has been demonstrated [20,21,22], as also the relevance of multilevel analyses and for intervention and organizational change studies [23,24,25,26,27,28,29]. 

As part of a research and development project for use in Swedish workplaces, several workplace surveys have been conducted in close collaboration with stakeholders from different organizations. The data and experiences from this process have contributed to the international development of COPSOQ III, e.g., selection of items, changes in wording and inclusion of new dimensions [5].

Now the Swedish standard version of COPSOQ III has been developed. As it is adapted to the Swedish context, it differs from the international version of COPSOQ III, which showed satisfactory basic psychometric properties in findings from 6 countries (including data collected at Swedish workplaces) [5,30]. The factor structure of the mandatory “core” items defined for COPSOQ III has been validated in Canada [31] and the COPSOQ III domain for Social Capital has been validated by qualitative and quantitative methods in Sweden [10,32]. 

Aggregated group means for organizations or departments are of high relevance for the assessment, implementation and evaluation of organizational interventions [33]. Although this approach is widely applied when applying COPSOQ for psychosocial risk management in workplaces, the emphasis of validation studies has so far been on the individual level. Nevertheless, a validation study is needed for the presentation and evaluation of the adapted Swedish national standard version of COPSOQ III, to establish population-based benchmarks for Sweden, and especially the aggregation to workplace group means has yet to be validated.

### A Need for Benchmarks for Use at Workplaces

Benchmarks can provide various kinds of relevant information for use at workplaces. Population-based benchmarks/reference values are the key to interpreting COPSOQ survey results from a risk management perspective [34]. For COPSOQ II, such population-based reference values are established, for example, for the working populations in Denmark, Spain, Canada, and France. For Sweden, the opportunities for comparisons have so far included mean scores from a convenience sample of workplace surveys (www.copsoq.se). Such comparisons can give an idea about the level for each scale for specific occupations but are not representative for the average level in the population. This forces occupational safety and health companies, organizational consultants, HR departments, policy-makers and researchers to interpret results from Swedish surveys with Danish reference values in order to assess psychosocial risks. This is not an ideal situation for several reasons: The data used for establishing the Danish reference values was collected 15 years ago [7]; the Danish labour market and legislation differs from the Swedish; the Danish benchmarks have not been validated for use in the Swedish context or with a Swedish language version; and finally the values relate to COPSOQ II. Introducing COPSOQ III accentuates the need for updated reference values based on the Swedish labour market of today.

The purpose of this study is to present and evaluate aspects of reliability and construct validity at both individual and workplace levels for the Swedish standard version of COPSOQ III, with the aim of establishing benchmarks for the organizational and social work environment for the adult working population in Sweden.

## 2. Materials and Methods

The present validation study builds on data from a cross-sectional national survey for the establishment of reference values and for psychometric evaluation of scale characteristics at the individual level. Nested data from a convenience sample of 51 workplace surveys is used for evaluation of the appropriateness of aggregating individual-level COPSOQ dimensions to the organizational level.

### 2.1. Random Sample

A cross-sectional survey was conducted by Statistics Sweden (SCB) at the request of the research group. Data collection took place from September to November 2018 by post, including an information letter, a paper version together with a stamped return envelope, and a personal link to a web questionnaire. Non-respondents received up to two reminders, the last of these included new paper questionnaires and return envelopes.

**Figure 1:**
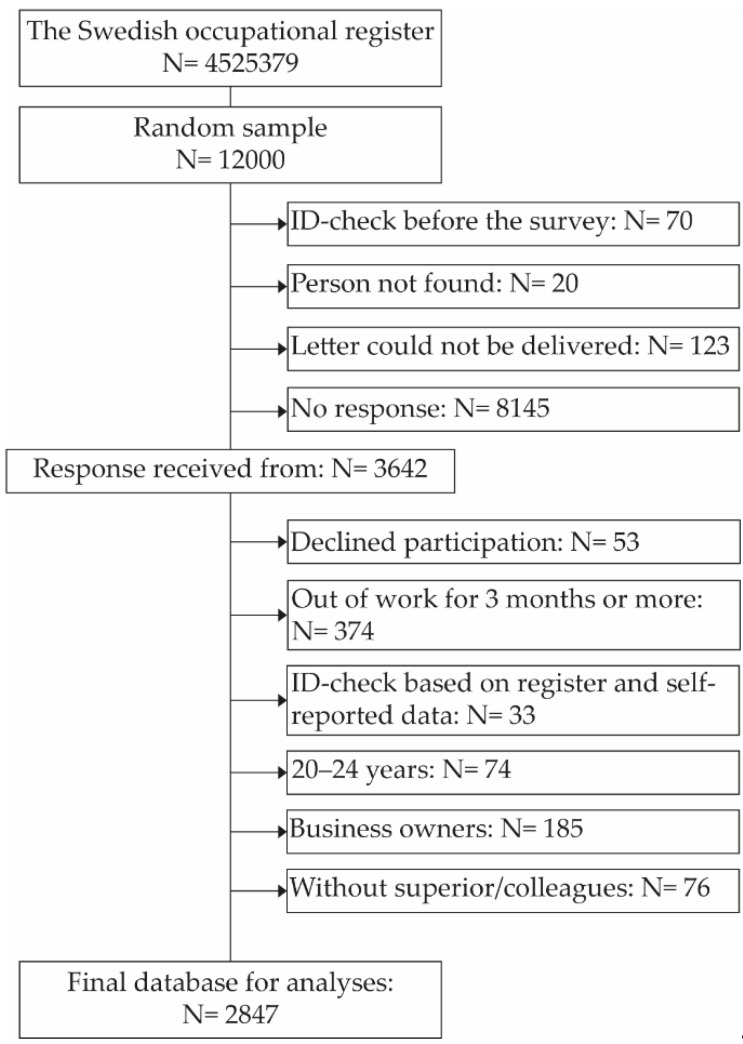
The selection process for the national random sample study. Inclusion criteria were 25–65-year-old workers living in Sweden, gainfully employed during the last 3 months before the survey and having a superior/colleagues.

From the Swedish employment directory, SCB drew a random sample of 11,556 persons from all 4,525,274 inhabitants in Sweden aged 20–65 years and registered as gainfully employed. In total, 3642 responded (30.9%). Of these, 53 declined participation, 374 were not currently in work, and 33 were excluded based on an ID-check comparing register data with self-reported data. Due to a response rate as low as 6% for those aged 20–24 years and the fact that many in this age group were still in education, we decided to exclude this age group (74 cases) from the analyses for this paper. In addition, 185 business owners and 76 respondents stating that they had neither a superior nor colleagues were excluded from all main analyses. For an overview of the sampling process, see Figure 1.

In general, women, the oldest age group, and those with tertiary education were the most likely to respond. This was also reflected in the differences seen across major occupational groups based on the International Standard Classification of Occupations, ISCO-08. People born in Scandinavia were more likely to respond than those born elsewhere, and those with the highest income responded to a larger extent than others. 

The study population is presented in Table 1. Out of the 2847 respondents in the analytic sample, 56% were women, the most frequent major occupational group was Professionals (group 2, 35%), and less than half of the respondents worked in the private sector (47%). Two out of three were in a non-managerial position (67%) and most respondents (81%) reported having direct contact with patients, customers, clients, pupils, etc., at work. More details regarding the study population stratified by major occupational groups (ISCO-08 1-digit) are presented in Table A1 (Appendix A).

### 2.2. Workplace Sample 

Cross-sectional data was collected from 2016 to 2019 as part of a validation and development project for the use of COPSOQ at workplaces (Grant: AFA Insurance 130301). All staff members in a convenience sample of 51 workplaces (organizations with max. 200 employees each; 26 public and 25 private) received an email with a link to an online questionnaire and an introduction and information about the research project. Each survey was open for 3–4 weeks and included two reminders. The overall response rate for the workplaces was 77% (ranging from 50% to 100%) and analyses included data from 1818 non-managerial employees. The average number of respondents at the workplaces was 28 (SD 18, range 8–138). For this convenience sample, 28% of the employees were under 35 years of age, 22% were 35–44, 27% were 45–54, and 21% were aged 55 or older and 51% were women. The corresponding distribution for the target population 2017 was according to SCB statistics: 26% below age 35, 26% were 35–44 years old, 28% were 45–54 and 21% were 55 or older and 48% were women. Most employees were Professionals (36% ISCO group 2), Technicians and Associate Professionals (24% ISCO group 3), Clerical Support Workers (11% ISCO group 4) or Services and Support Workers (12% ISCO group 5).

### 2.3. Variables

The questionnaire for the national study comprised 132 items in total and a free text field for comments. We included 12 background factors regarding work situation and personal characteristics in addition to register data obtained from Statistics Sweden. From COPSOQ III, 85 items were included in the questionnaire to cover 33 dimensions. Furthermore, 35 items were included for other research purposes. The questionnaire applied to employees at workplaces was regarding COPSOQ III items similar to the questionnaire used for the national survey. 

### 2.4. The National Swedish Standard Version of COPSOQ III

In the present study, we evaluate the national Swedish standard version of COPSOQ III. It includes 76 items (according to the international COPSOQ III structure: 32 mandatory “core” items, 15 additional “middle” items and 29 additional “long” items) to cover 33 work environment dimensions (24 multi-item scales, nine single item measures (incl. five items on conflicts and offensive behaviours). Table A2 from Appendix B gives an overview of the Swedish standard version of COPSOQ III and its correspondence with the international middle version of COPSOQ III and with the Swedish middle version of COPSOQ II. A detailed overview, including formulations in Swedish, is available as an online Supplementary Materials. In relation to the previous Swedish version, the present third version includes five new dimensions and six dimensions have changed name, one dimension has changed response options, 16 dimensions have a reduced number of items, two items are replaced and five have changes in wording. Decisions regarding the selection of dimensions were guided by the perceived relevance to the Swedish context, cognitive interviews, pilot tests and dialogue with stakeholders, taking the item level in the international COPSOQ III and item-level ICC(1) values into consideration for not jeopardizing the ability to differentiate workplaces, as recently suggested by Bliese and colleagues [35].

### 2.5. Analyses

Scales were computed as means of items with range 0–100, where the scale score was set to missing if respondents had replied to less than half of the items included in the scale [5]. Each scale was scored in the direction indicated by its name [5].

To draw correct inferences about the target population, two sets of weights were calculated for the national representative sample; one based on sex, age, income and educational level for calculating benchmarks for the general population of 25–65-year-old employees in Sweden; and another set of weights based on sex and age for the purpose of calculating representative mean scores for each of the ISCO major occupational groups. The benchmarks for the Swedish standard version of COPSOQ III were computed as mean scores with standard deviations for scales, and frequencies of conflicts and offensive behaviours such as bullying, harassment and violence based on weighted data to match the target population of 25–65-year-old employees working in Sweden. Mean scale scores, standard deviation and frequency of conflicts and offensive behaviours were also computed for each major occupational group, weighted within each group to match the target population (ISCO 1-digit, 25–65 years). Internal consistency reliability was analysed with Cronbach’s alpha for scales with three or more items and Spearman-Brown Coefficient for two-item scales [36]. The proportion of respondents selecting the lowest (floor) and highest (ceiling) response options for all items in a scale were determined for all scales, as well as the proportion of respondents having replied to less than half of the items in each scale (scale missing). More than 15% of the respondents choosing the lowest or highest response options was considered evidence of a floor or ceiling effect, respectively [37]. Mean scores and frequency of conflicts and offensive behaviours were calculated according to sex (men/women), work sector (private/public) and white/blue-collar work (ISCO groups 1–2–3 versus 6–7–8–9). Differences within each group were tested with t-tests and Chi-square tests, and Cohen’s d was calculated for evaluation of the effect of sex, sector, and kind of work. A Cohen’s d value of 0.2 indicates a small effect, 0.5 a medium effect and 0.8 a large effect [38] and a 5–10 point mean score difference is considered a minimum important difference [39].

ICC(1) and ICC(2) were calculated for each dimension based on aggregation of individual level data to ISCO major occupational group (national sample) and to workplace (workplace sample). ICC(1) represents the amount of variance in the employees’ responses that can be explained by their membership of a group (occupation or workplace) [35,40,41,42]. ICC(1) values of 0.05 can be considered as a small to medium effect and higher values indicate stronger effects [42], ICC(2) is an estimate of reliability of the aggregated group means [35,40,41]. Values <0.5 indicate poor reliability, 0.5–0.75 moderate and >0.75 indicate good reliability of group-level means [43]. Finally, for the sample of workplaces, we calculated the aggregated level mean, standard deviation, minimum, maximum, range and comparison of mean scores with the Benchmark for each scale.

Bivariate Pearson correlations between scales were calculated for the national sample of 25–65-year-old employees (individual level) and for the convenience sample of workplaces (individual and workplace level) for evaluation of construct validity (distinctiveness of dimensions and concurrent validity). 

### 2.6. Ethics

Informed consent was obtained from all individual participants included in the study. All procedures performed were in accordance with the ethical standards of the national research committee and with the 1964 Helsinki Declaration and its later amendments or comparable ethical standards. The Regional Ethical Review Board of Sweden approved the study (Dnr 2015-476; 2018–392; 2019-05904).

## 3. Results

Benchmarks for the Swedish standard version of COPSOQ III are presented in Table 2 in addition to scale psychometric characteristics. 

The internal consistency reliability was above 0.70 for all scales, except for the two-item scale for Quality in Work (0.69). Most dimensions had low floor and ceiling effects. High floor effect and low mean scores were seen for Job Insecurity (34.8%) and Insecurity over Working Conditions (28.1%). A strong ceiling effect and high mean values were seen for the single item Meaning of Work (40.6%) and for Social Support from Supervisor (30.3%) and Social Support from Colleagues (32.5%). Internal non-response for dimensions was between 0.4% and 1.6%.

The mean scores differed statistically significantly for most scales by sex, work sector and white/blue-collar work (Table 3).

Moderate to large differences in mean scores were found between white- and blue-collar workers, in particular. White-collar workers had higher mean scores for Quantitative Demands, Emotional Demands, Influence, Possibilities for Development, Variation and Meaning of Work, while lower for Job Insecurity compared to blue-collar workers. Emotional Demands was the only dimension showing large differences for sex, work sector and kind of work. Women workers, employees working in the public sector and white-collar workers reported the highest levels of Emotional Demands (scale means 15–19 points higher than for their respective counterparts). We found a corresponding pattern with the same groups most exposed to conflicts and offensive behaviours. An additional comparison revealed that business owners scored statistical significantly higher for the outcome dimensions Work Engagement (77) and Job Satisfaction (72), and lower for Stress (31) and Burnout (31) than the study sample did (results not shown in table).

Table 4 displays psychometric characteristics for major occupational groups based on the ISCO-08 classification. Of the 24 relevant scales, 16 revealed satisfactory reliability values for all major occupational groups. Reliability coefficients below 0.70 were mainly seen among Managers and Elementary Occupations (e.g., Work pace, Recognition, Role conflicts and Quality in work), and only in one case did a reliability coefficient reach below 0.60 (Work Pace/Elementary Occupations). Managers reported the most beneficial scores across occupations (14 out 28 scales) and the group having the most problematic weighted mean scores was Plant and Machine Operators and Assemblers (13 out of 28 scales). Services and Support Workers was the group most exposed to Threats of Violence, Physical Violence and Sexual Harassment. Clerical Support Workers reported bullying most frequently, while Managers were the group most exposed to Cyber Bullying. The widest range for mean scores across ISCO major occupational groups was found for Emotional Demands, Variation, Quantitative Demands and Influence.

The bivariate intercorrelations between dimensions for the total national sample (individual level data) are presented in Table 5 and for the workplace sample (both individual and organizational level) in Table 6. Too strong intercorrelations may indicate that the scales do not measure distinct constructs. For individual level data, only 6 out of the 378 correlations in the national sample and 9 correlations in the workplace sample were above 0.70. The strongest correlations at the individual level were largely those between scales that were most strongly correlated also at the workplace level, for example, the correlation between Stress and Burnout ranged from 0.79 to 0.83. The correlations were in general stronger between scales aggregated to the organizational level than the corresponding correlations at the individual level. Nevertheless, for the scales Role Clarity and Quantitative Demands, most of the correlations with other dimensions were strongest at individual level. We found differences in the pattern of correlations between individual and workplace level data in relation to a few dimensions, in particularly Role Clarity and Job Insecurity. For example, a moderate negative correlation was seen between Job Insecurity and Quantitative Demands (−0.53) at an organizational level, while the corresponding correlation was non-significant at an individual level. Conversely, a moderate positive correlation between Role Clarity and Social Community at Work was significant at an individual level (0.37/0.34) but insignificant at a workplace level.

Table 7 displays measures relating to aggregation of data to major occupational groups and to organizational level. The ICC(2) scores indicate a moderate to good reliability of group mean scores for major occupational groups as well as for workplaces. Only aggregation of the individual characteristic Self-Rated Health to workplace level showed poor reliability. A small to medium effect of respondents’ major occupational group was seen for Quantitative Demands, Emotional Demands, Influence, Possibilities for Development, Variation, Meaning of Work, and in addition for Job Insecurity (ICC(1)). In relation to the effect of workplace, the largest explained variance was seen for scales reflecting job demands and aspects of leadership, while small to medium effect sizes were found for all other exposures. The aggregated workplace mean scores ranged from 23 to 54 points. 

## 4. Discussion

In the present study, we have evaluated the reliability and construct validity of a Swedish standard version of COPSOQ III at both individual and organizational level and established national benchmarks for workplace surveys. A trade-off exists between the obvious need for a questionnaire of high relevance for the national context and the need to keep a high degree of correspondence with other national versions for facilitation of valid comparisons. Experiences from previous versions of the instrument have shown that practitioners and researchers to a high extent share a wish for shorter questionnaires. We have chosen to reduce the number of items in many dimensions in order to be able to make room for new dimensions covering Work Engagement, Quality of Work, Job Insecurity, Insecurity over Working Conditions and Cyber Bullying. Scales including only a few items potentially reduce the reliability and validity of the measurement. Nevertheless, our overall findings indicate that the Swedish national standard version of COPSOQ III has good psychometric properties for its intended uses. 

### 4.1. Reliability and Scale Characteristics at Individual Level Based on the National Survey

The internal consistency reliability of the scales was satisfactory for the study population as a whole. This corresponds with findings from the international COPSOQ III validation study (Burr et al. 2019). An unacceptably low value for Work Pace was seen for respondents with an Elementary Occupation, and the reliability was questionable for Work Pace, Role Conflicts and Quality of Work for Managers, Craft and Related Trade Workers, and Elementary Occupations. This calls for caution when interpreting results for these specific combinations of scales and major occupational groups. In the future, adding more items to these scales should be considered in the Swedish context. 

Compared to findings from the Danish COPSOQ II study [7] and the international COPSOQ III study [5], the internal non-response was low for all scales, and especially regarding Social Support from Supervisor and Vertical Trust. Scales referring to managers and work climate can in some cases be difficult to reply to, for example in complex organizations or among the self-employed [10]. The noticeable lower internal non-response for these scales might be due to stricter inclusion criteria in the present study in combination with the thorough adaptation of formulations based on cognitive interviewing techniques [8,9,10]. 

Floor and ceiling effects were minor for most scales, indicating the good ability of the instrument to distinguish over the full spectrum of the scales. However, for the new dimensions, Job Insecurity and Insecurity over Working Conditions, we found a high floor effect. This finding was not a surprise based on the previous findings from the international validation study [5] and from the Sixth European Working Conditions Survey [44]. In contrast, we found large ceiling effects for Meaning of Work, Social Support from supervisor and from colleagues. The finding regarding Meaning of Work is also in accordance with previous findings [5,11]. Sweden is globally among those countries with the highest proportion of workers employed in service work (2019: 80% [45]), which is typically perceived as more meaningful than manufacturing work. The high levels of reported social support contrast with the levels reported for COPSOQ II for specific occupational contexts in Sweden [11,16]. This could be a consequence of the COPSOQ III standard version including two rather than three items in each of these scales. The level is also higher than the reported international results reported for COPSOQ III [5]. This difference can probably be understood in the light of the Swedish workplace culture characterized by shared decision making, avoidance of conflicts and aiming at consensus [46]. 

### 4.2. Reliability and Validity of COPSOQ III for Use at Workplaces and for Multilevel Research Design

COPSOQ is a generic instrument intended for research purposes as well as risk management of the psychosocial work environment at workplaces [5,7,47]. Accordingly, the ability of scales to distinguish exposures for different occupational groups and across workplaces is of great importance.

Despite being an instrument, which collects responses from individual employees, the main intention is to capture workplace and organizational conditions, not individual perceptions. It is thus very important that the aggregated workplace scores refer to something that is shared by the employees in a certain work unit/organization and not just to a mean of largely unrelated individual responses. Our findings corroborated the reliability of such group mean scores regarding psychosocial exposures based on aggregation to occupation and workplace level. 

The traditional criterion is a minimum of 5% explained variance for the relevance of taking the aggregated level into account [41,42]. The amount of variance explained by workplace fulfilled the criteria for all dimensions except Self-Rated Health, which is an individual outcome mainly influenced by non-work-related factors. This underlines the importance of considering the workplace level for research on the psychosocial environment and justifies the relevance of aggregating individual scores to group mean scores when reporting survey results back to workplaces. Our findings corroborate previous research on the COPSOQ II showing that job exposure matrices are of little relevance for psychosocial risk assessment of, e.g., relational factors in workplaces [48,49]. However, the low amount of variance attributed to the major occupational groups does not imply that occupation is of no relevance, as the ISCO-digit-1 grouping comprises many different occupations working in different sectors, etc., within each major group. In a specific context such as public dental services, psychosocial work environment factors have been reported to differ considerably for dentists working in different organizations, while this is not the case for dental nurses and hygienists [22]. Additionally, the traditional criterion has been questioned as even ICC(1) values as low as 0.01 in some cases are relevant to take into account in multilevel analyses [50].

We found a similar overall pattern of inter-correlations at the individual level across the two samples of the present study and those reported from the international validation study [5] (Burr et al. 2019). In general, the strength and direction of correlations supported the concurrent validity of the scales. However, the strength of the inter-correlation between Stress and Burnout and the similarity of correlation for these two scales to other dimensions calls for further clarification of whether they actually represent two separate constructs as measured here. 

As one might expect, however, we found differences in the strength of correlations at the individual level when comparing the Swedish with the international findings. In particular, the two new dimensions regarding insecurity showed considerably stronger correlations with other dimensions in the Swedish sample compared to the international average correlations across national samples. A high degree of employment security on the labour market combined with a high flexibility decreases the detrimental health effect of individual employees’ perceptions of job insecurity [51]. The Swedish labour market is, however, characterized by high employment security for people in fixed positions, but little flexibility in hiring and firing of workers; this combination may result in especially strong adverse reaction to individual level experienced job insecurity [51]. 

In accordance with what is typically reported [52], the correlations at the aggregated workplace level were in general stronger than for the individual level. We found some interesting differences in the general pattern of correlations between individual and workplace level. This may be due to conceptual differences between aggregated and individual level dimensions [33]. Stronger correlations at the individual level could also indicate individual bias, such as negative affectivity or generalized effects of health, for instance depressive symptoms [53]. Stronger correlations at the organizational level, on the other hand, could indicate generalized effects of managerial practices or financial constraints at the organizational level. For example, the Psychosocial Safety Climate (PSC) of organizations has been shown to act as a precursor to and moderator of job demands and resources in the workplace [54,55,56]. This underlines the importance of careful theoretical considerations and the relevance of multilevel study design in work environment research in order to avoid the ecological or the atomistic fallacy. 

### 4.3. Strengths and Limitations 

The findings of our study should be seen in the light of some advantages and limitations. 

A trade-off exists between the need to optimize the relevance of a generic questionnaire to the local context and the prospects for comparison over time and context. We found it to be possible to reduce the number of items, to maintain a broad coverage and even include new dimensions of high relevance to Swedish regulations (e.g., Work Engagement and Quality of Work). Another advantage is that the Swedish national version of COPSOQ III builds on experiences from COPSOQ II and a careful adaptation process including translation-back-translation, use of cognitive interviews and perceptions from stakeholders of different kinds.

The study design allowed for analyses including individual level data and nested data from workplaces. This adds to the knowledge about the reliability and validity of the instrument for use at workplaces and for integration in multilevel analyses. 

The response rate for the workplace sample was a satisfactory 77%, clearly indicating the relevance of the instrument for use in this context. For the national survey the response rate was a less satisfying 31% and for two of the major ISCO 1-digit groups the number of respondents was too low to allow for valid calculation of scale mean scores. However, the strength of this dataset is that it was based on a random sample of wage earners in Sweden and the opportunity of calculating weights for adjustment based on complementary demographic register data. A comparison of weighted and unweighted benchmarks and mean scores (not reported) showed only minor differences in estimates. While the low response rate still is a limitation of the study, we find no indication that selection bias is a major problem for the reported population-based benchmarks and mean values for the major occupational groups, which can thereby be considered representative of the underlying population. 

In future studies, it will be relevant to employ a longitudinal multilevel design with integration of self-reported data and register data (e.g., absence, staff turnover and measures of performance). In particularly, it will be relevant to evaluate test-retest reliability, responsiveness and predictive criterion validity. Bliese and Jex pointed out that simple analyses of means for people working together often may be appropriate for implementation and evaluation of organizational interventions and are also important to consider in stress research projects [33]. This makes further validation of the multilevel structure of the instrument and evaluation of measurement invariance across different groups and language versions highly relevant. 

## 5. Conclusions

The present study supports the reliability and construct validity of the Swedish standard version of COPSOQ III and establishes benchmarks for workplace risk management as well as for research purposes.

## Figures and Tables

**Table 1:** Description of respondents based on a random sample of inhabitants in Sweden aged 25–65 years, gainfully employed (N = 2847).

Dimension	Group	% of Sample
Sex	Men	43.9
	Women	56.1
Age, mean (SD)	47.7 (10.8) years	
Age distribution	25–39 years	25.1
	40–54 years	42.9
	55–65 years	32.0
Occupational group	0. Armed Forces Occupations	0.20
	1. Managers	7.80
	2. Professionals	34.7
	3. Technicians and Associate Professionals	14.8
	4. Clerical Support Workers	7.70
	5. Services and Support Workers	16.9
	6. Skilled Agricultural. Forestry and Fishery Workers	0.70
	7. Craft and Related Trades Workers	5.90
	8. Plant and Machine Operators and Assemblers	5.30
	9. Elementary Occupations	3.10
	Not classified	2.90
Educational level	Primary education	5.10
	Secondary education	45.5
	Tertiary education	49.5
Income level	Up to 300,000 Sek	25.9
	300,001–400,000 Sek	31.6
	More than 400,000 Sek	42.5
Region of birth	A Scandinavian country	90.8
	Other countries	9.20
Sector	Private	47.1
	Public	44.7
	Other	5.60
	Not stated	2.60
Weekly work hours	<21	3.90
	21–30	5.40
	31–40	55.1
	41–50	30.7
	>50	2.80
	not stated	2.10
Work situation	Have direct contact with patients. customers, clients, pupils etc. at work	81.1
	Non-managerial position	66.8
Normal work time	Day hours between 6–18 o’clock	78.5
Size of local workplace (span of nearest leader)	<5 people	14.3
	5–10 people	22.4
	11–20 people	25.1
	21–40 people	22.5
	41–60 people	6.90
	>60 people	5.20
	Do not know/not stated	3.60

**Table 2:** For the Swedish standard version of COPSOQ III for 25–65-year-old workers in Sweden: Benchmarks with standard deviations (SD) and frequency of conflicts and offensive behaviours (based on weighted data) and scale characteristics (number of items, reliability coefficient, floor, ceiling and scale missing percentages, based on raw data).

Population Benchmarks				Scale Characteristics				
Dimension and Abbreviation		Mean	SD	No. of Items	Reliability Coefficient ^1^	Floor %	Ceiling %	Scale Missing %
Quantitative Demands	QD	40.9	22.1	3	0.85	4.9	1.1	1.2
Work Pace	WP	59.9	20.5	2	0.70	0.6	4.9	0.9
Emotional Demands	ED	46.8	25.5	3	0.86	3.9	1.5	1.1
Influence	IN	50.2	20.1	4	0.75	0.9	0.6	1.1
Possibilities for Development	PD	70.4	20.0	3	0.75	0.4	10.1	0.4
Variation of Work	VA	68.0	22.5	1		1.9	16.7	1.1
Meaning of Work	MW	78.3	22.4	1		0.9	40.6	1.6
Predictability	PR	60.2	21.3	2	0.73	1.5	5.3	1.1
Recognition	RE	65.6	23.3	2	0.74	2.0	11.2	1.1
Role Clarity	CL	78.1	16.7	3	0.78	0.0	16.1	0.2
Role Conflicts	CO	42.2	19.6	3	0.71	2.0	0.5	0.6
(incl. illegitimate tasks)								
Quality of Leadership	QL	54.1	24.8	3	0.87	4.6	4.8	0.9
Social Support from Supervisor	SS	75.3	24.5	2	0.88	1.8	30.3	0.4
Social Support from Colleagues	SC	80.2	19.6	2	0.81	0.4	32.5	0.4
Sense of Community at Work	SW	79.9	15.0	3	0.78	0.1	17.7	0.4
Commitment to the Workplace	CW	64.7	24.5	3	0.83	1.1	9.8	1.2
Work Engagement	WE	69.4	19.2	3	0.84	0.4	5.4	0.9
Job Insecurity	JI	20.2	20.9	3	0.75	34.8	0.4	1.1
Insecurity over Working Conditions	IW	24.9	23.2	2	0.77	28.1	1.4	1.1
Quality of Work	QW	68.2	18.6	2	0.69	0.6	6.6	0.4
Job Satisfaction	JS	64.4	20.2	4	0.84	0.7	5.2	0.8
Work Life Conflict	WF	39.7	25.7	3	0.90	9.5	3.6	0.6
Horizontal Trust	TE	71.3	20.1	1		1.2	17.6	1.4
Vertical Trust	TM	69.3	19.0	3	0.77	0.4	7.0	0.9
Organizational Justice	JU	59.7	20.2	3	0.78	0.8	3.5	0.9
Self-Rated Health	GH	61.3	23.9	1		2.6	12.8	1.0
Stress	ST	36.0	24.2	3	0.86	11.2	1.3	1.2
Burnout	BO	36.2	24.7	3	0.88	10.2	1.4	1.0
Threats of Violence	TV	10.5%		1				1.1
Physical Violence	PV	5.3%		1				1.4
Bullying	BU	10.3%		1				1.4
Sexual Harassment	SH	6.0%		1				1.2
Cyber Bullying	HSM	2.7%		1				1.2

^1^ Cronbach’s alpha for scales with 3 or more items and Spearman-Brown Coefficient for two-item scales.

**Table 3:** Scale mean scores and frequency of conflicts and offensive behaviours according to sex, work sector and white/blue-collar work for the Swedish standard version of COPSOQ III for 25–65-year-old workers in Sweden. Differences between groups tested with t-tests, Chi^2^ tests ^1^ and Cohen’s *d*.

Dimension ^2^	Sex				Work Sector				Kind of Work			
	Men (*n* = 1250)	Women (*n* = 1597)	*p*	Cohen’s *d*	Private Sector (*n* = 1341)	Public Sector (*n* = 1271)	*p*	Cohen’s *d*	White Collar (*n* = 1630)	Blue Collar (*n* = 428)	*p*	Cohen’s *d*
QD	41.2	43.2	*	0.1	40.6	44.3	**	0.2	47.7	33.0	**	0.7
WP	57.8	60.9	**	0.2	59.7	59.6		0.0	60.1	58.0		0.1
ED	39.4	54.1	**	0.6	38.5	57.1	**	0.8	49.9	33.2	**	0.7
IN	53.9	48.6	**	0.3	52.4	49.2	**	0.2	54.6	46.1	**	0.5
PD	70.2	71.7	*	0.1	70.0	72.5	**	0.1	75.4	62.6	**	0.7
VA	67.7	71.3	**	0.2	67.9	71.8	**	0.2	74.1	59.6	**	0.7
MW	76.0	82.2	**	0.3	74.5	84.9	**	0.5	81.8	70.5	**	0.6
PR	60.3	60.9		0.0	60.4	61.2		0.0	61.6	58.0	*	0.2
RE	66.9	64.9	*	0.1	66.6	65.0		0.1	67.8	62.5	**	0.2
CL	76.8	78.9	**	0.1	77.7	78.7		0.1	76.5	79.6	**	0.2
CO	42.5	41.3		0.1	40.1	43.9	**	0.2	43.0	39.8	*	0.2
QL	54.0	54.1		0.0	54.3	53.8		0.0	54.9	51.6	*	0.1
SS	75.2	75.0		0.0	76.6	73.3	**	0.1	75.8	72.1	*	0.2
SC	79.2	81.3	*	0.1	79.9	81.0		0.1	81.4	77.0	**	0.2
SW	80.1	80.0		0.0	81.1	79.1	**	0.1	80.6	79.1		0.1
CW	65.6	65.2		0.0	65.8	65.0		0.0	66.9	62.4	**	0.2
WE	68.3	71.9	**	0.2	69.0	71.5	**	0.1	71.8	65.0	**	0.4
JI	20.3	17.8	**	0.1	21.7	14.9	**	0.3	15.2	26.3	**	0.6
IW	23.9	24.5		0.0	23.8	24.7		0.0	22.6	26.2	*	0.2
QW	68.5	67.4		0.1	69.4	66.2	**	0.2	67.6	69.9	*	0.1
JS	65.3	64.8		0.0	65.8	64.5		0.1	67.2	61.6	**	0.3
WF	37.5	41.7	**	0.2	38.2	41.6	**	0.1	42.0	35.9	**	0.2
TE	72.5	70.7	*	0.1	72.3	70.5	*	0.1	73.5	68.3	**	0.3
TM	68.5	70.2	*	0.1	69.9	69.3		0.0	70.6	66.5	**	0.2
JU	60.6	58.5	*	0.1	61.0	57.8	**	0.2	60.6	57.9	*	0.1
GH	63.2	60.6	*	0.1	63.3	60.2	**	0.1	63.9	58.2	**	0.2
ST	32.6	37.7	**	0.2	33.9	36.9	*	0.1	36.8	31.7	**	0.2
BO	31.2	38.1	**	0.3	32.9	37.4	**	0.2	34.4	33.9		0.0
TV	8.1%	12.5%	**		5.3%	16.4%	**		9.6%	6.9%		
PV	2.7%	7.5%	**		1.7%	9.4%	**		4.2%	1.4%	*	
BU	8.4%	11.8%	*		9.1%	11.4%	*		9.0%	10.1%		
SH	2.4%	6.8%	**		4.5%	5.1%			4.2%	4.0%		
HSM	2.8%	2.8%			1.7%	3.8%	**		3.3%	1.4%	*	

^1^ * *p* < 0.05 level, ** *p* < 0.001 level. ^2^ Abbreviations of dimensions explained in Table 2.

**Table 4:** Mean scale scores and standard deviation and frequency of conflicts and offensive behaviours according to occupational groups for the Swedish Standard version of COPSOQ III (aged 25–65 years, weighted within ISCO major occupational groups). Reliability Coefficients ^2^(RC) based on unweighted data.

Dimension ^1^	ISCO 1 Managers			ISCO 2 Professionals			ISCO 3 Technicians and Associate Professionals			ISCO 4 Clerical Support Workers			ISCO 5 Services and Support Workers			ISCO 7 Craft and Related Trades Workers			ISCO 8 Plant and Machine Operators and Assemblers			ISCO 9 Elementary Occupations		
	Mean	SD	RC	Mean	SD	RC	Mean	SD	RC	Mean	SD	RC	Mean	SD	RC	Mean	SD	RC	Mean	SD	RC	Mean	SD	RC
QD	51.5	19.8	0.86	48.5	20.0	0.82	43.8	20.4	0.84	39.7	21.6	0.83	34.5	22.1	0.85	36.0	20.3	0.83	32.0	19.6	0.82	27.8	20.2	0.80
WP	63.3	18.0	0.68	60.5	18.7	0.70	57.6	18.7	0.71	57.1	20.3	0.72	60.0	21.9	0.76	58.1	18.4	0.62	59.0	21.5	0.74	58.6	23.2	0.46
ED	53.7	20.6	0.82	53.1	25.5	0.87	40.3	23.1	0.82	37.4	24.2	0.85	59.5	22.8	0.82	31.4	18.4	0.78	34.7	21.0	0.77	34.4	22.5	0.72
IN	62.1	17.0	0.74	53.8	17.5	0.73	52.8	17.6	0.71	43.7	20.2	0.77	45.2	20.1	0.73	49.5	18.8	0.72	41.0	22.4	0.83	45.8	21.6	0.75
PD	79.3	15.7	0.72	76.3	17.1	0.73	71.1	18.1	0.74	62.8	21.9	0.77	67.4	19.1	0.72	66.2	19.2	0.69	58.5	22.4	0.74	61.4	21.5	0.72
VA	79.4	15.3		74.8	18.0		69.9	18.6	-	62.3	23.3	-	67.0	22.8	-	66.7	20.9	-	53.9	27.5	-	54.7	27.3	-
MW	83.3	16.0		83.6	18.9		76.9	19.7	-	72.7	25.1	-	83.5	20.2	-	71.2	23.5	-	67.7	24.7	-	72.7	25.0	-
PR	68.4	19.1	0.66	60.8	19.8	0.72	60.0	20.0	0.70	58.4	20.4	0.69	61.3	21.2	0.76	56.2	20.4	0.75	57.1	24.0	0.81	62.1	23.8	0.73
RE	73.2	19.2	0.68	66.9	21.6	0.70	66.9	22.1	0.73	62.1	23.8	0.79	63.3	24.4	0.77	62.9	22.4	0.69	60.6	25.5	0.78	63.1	24.7	0.66
CL	78.7	15.2	0.77	76.0	17.0	0.79	76.4	17.3	0.81	77.4	16.1	0.74	82.8	14.3	0.76	77.3	16.6	0.79	80.7	14.3	0.64	81.9	17.3	0.77
CO	41.9	16.2	0.61	43.9	19.1	0.71	41.3	19.1	0.72	37.4	18.5	0.65	42.1	20.0	0.70	40.7	17.5	0.65	40.6	22.9	0.82	37.7	21.2	0.64
QL	57.7	22.1	0.84	54.3	22.9	0.84	55.0	25.2	0.87	52.4	26.1	0.88	54.0	25.9	0.90	48.7	24.3	0.86	50.4	25.7	0.87	58.1	24.9	0.88
SS	77.7	20.9	0.81	75.2	23.5	0.88	76.0	24.6	0.89	76.7	23.5	0.85	75.1	24.9	0.89	71.4	25.5	0.82	70.3	27.7	0.89	73.3	27.0	0.95
SC	79.6	9.9.2	0.76	81.8	18.3	0.82	81.6	18.0	0.79	78.4	19.5	0.79	80.8	19.1	0.83	79.5	18.2	0.81	74.5	23.3	0.83	75.9	22.9	0.81
SW	82.3	12.8	0.76	80.1	14.0	0.76	81.1	14.5	0.79	78.3	16.0	0.80	79.7	15.2	0.80	80.3	13.0	0.74	76.3	17.8	0.83	81.2	16.4	0.77
CW	73.3	22.1	0.85	65.7	22.9	0.82	66.5	23.8	0.83	62.8	25.7	0.84	63.7	25.4	0.83	62.8	21.8	0.76	60.8	24.7	0.82	62.2	25.4	0.81
WE	75.2	16.6	0.86	71.8	16.8	0.82	69.9	18.0	0.85	67.0	20.1	0.82	71.4	18.7	0.84	64.3	19.7	0.85	64.1	21.7	0.85	66.5	21.4	0.87
JI	17.2	18.6	0.79	12.7	16.7	0.73	20.0	19.7	0.71	27.3	21.5	0.73	20.8	22.4	0.73	21.0	20.0	0.71	29.3	24.0	0.76	31.5	24.4	0.74
IW	19.8	21.5	0.85	22.6	21.5	0.75	24.0	22.9	0.80	25.5	22.6	0.78	27.5	24.1	0.71	22.4	21.4	0.81	29.8	26.3	0.82	29.7	26.6	0.81
QW	70.1	14.9	0.64	66.6	17.5	0.68	68.5	18.4	0.72	66.6	19.5	0.74	67.2	19.3	0.69	69.0	16.6	0.74	69.5	18.9	0.64	71.6	19.3	0.63
JS	72.7	18.0	0.84	66.6	18.9	0.81	65.6	19.0	0.84	62.0	20.2	0.85	61.8	20.6	0.86	62.9	17.1	0.83	60.4	21.2	0.91	60.4	21.1	0.88
WF	44.5	24.7	0.92	43.7	24.9	0.90	36.7	23.8	0.90	36.5	24.6	0.90	38.3	27.4	0.91	35.3	23.7	0.88	39.7	27.0	0.90	32.0	25.7	0.89
TE	72.3	18.0	-	73.3	17.7	-	74.4	18.9	-	67.0	20.9	-	69.2	20.4	-	68.9	19.2	-	67.0	24.5	-	70.1	20.7	-
TM	74.8	14.7	0.74	70.2	18.0	0.77	69.4	19.0	0.81	67.5	18.5	0.77	69.0	18.5	0.72	63.9	18.8	0.74	65.8	20.6	0.79	70.5	19.1	0.64
JU	67.5	15.3	0.74	59.3	17.8	0.74	60.1	19.1	0.79	55.1	21.1	0.79	58.5	21.4	0.79	55.8	20.0	0.78	57.7	22.9	0.84	60.2	22.6	0.78
GH	67.4	22.1	-	62.3	22.9	-	65.7	23.0	-	59.5	24.4	-	57.5	24.6	-	59.5	23.0	-	56.4	25.1	-	59.8	25.5	-
ST	36.8	20.9	0.81	38.3	24.0	0.87	33.2	24.4	0.87	35.0	23.7	0.86	34.8	25.4	0.87	31.4	22.6	0.84	32.0	24.3	0.87	33.3	24.6	0.81
BO	29.5	20.3	0.83	37.0	23.4	0.87	30.7	23.7	0.87	35.1	25.0	0.89	39.1	26.2	0.90	31.6	22.4	0.85	34.9	25.9	0.91	36.9	26.0	0.88
TV	8.2%			10.5%			8.2%			8.2%			18.9%			2.4%			11.3%			8.1%		
PV	2.3%			5.0%			3.4%			2.3%			15.2%			0.0%			2.0%			3.6%		
BU	5.0%			9.6%			9.6%			14.2%			12.5%			9.6%			8.8%			14.5%		
SH	1.4%			4.6%			4.8%			2.3%			9.3%			3.0%			3.3%			8.2%		
HSM	6.4%			3.5%			1.2%			4.1%			2.1%			1.2%			1.3%			2.4%		

^1^ Abbreviations of dimensions explained in Table 2. ^2^ Cronbach’s alpha for scales with 3 or more items and Spearman-Brown Coefficient for two-item scales.

**Table 5:** Pearson correlations between scales for the Swedish Standard version of COPSOQ III (national sample of 25–65-year-old employees).

Dimension ^1^	QD	WP	ED	IN	PD	VA	MW	PR	RE	CL	CO	QL	SS	SC	SW	CW	WE	JI	IW	QW	JS	WF	TE	TM	JU	GH	ST	BO
QD	1.00																											
WP	0.40	1.00																										
ED	0.29	0.32	1.00																									
IN	−0.03	−0.11	−0.09	1.00																								
PD	0.06	0.02	0.05	0.51	1.00																							
VA	0.13	0.02	0.12	0.37	0.50	1.00																						
MW	−0.02	0.03	0.21	0.27	0.50	0.36	1.00																					
PR	−0.21	−0.15	−0.13	0.39	0.43	0.20	0.34	1.00																				
RE	−0.19	−0.15	−0.16	0.48	0.49	0.27	0.35	0.67	1.00																			
CL	-0.26	0.04	−0.01	0.18	0.32	0.10	0.41	0.46	0.38	1.00																		
CO	0.41	0.30	0.36	−0.22	−0.20	−0.10	−0.22	−0.44	−0.41	−0.32	1.00																	
QL	−0.18	−0.12	−0.12	0.35	0.42	0.20	0.29	0.62	0.64	0.35	−0.38	1.00																
SS	−0.19	−0.15	−0.17	0.38	0.41	0.20	0.25	0.55	0.62	0.35	−0.36	0.67	1.00															
SC	−0.15	−0.15	−0.10	0.32	0.39	0.23	0.29	0.40	0.43	0.31	−0.29	0.38	0.58	1.00														
SW	−0.18	−0.09	−0.16	0.36	0.39	0.22	0.28	0.45	0.51	0.37	−0.33	0.42	0.48	0.62	1.00													
CW	−0.24	−0.19	−0.17	0.46	0.54	0.32	0.47	0.65	0.72	0.41	−0.50	0.62	0.56	0.46	0.54	1.00												
WE	−0.05	0.07	0.10	0.36	0.54	0.35	0.55	0.43	0.44	0.39	−0.27	0.39	0.31	0.28	0.37	0.58	1.00											
JI	−0.01	0.03	−0.09	−0.18	−0.32	−0.26	−0.28	−0.20	−0.26	−0.17	0.12	−0.15	−0.17	−0.23	−0.22	−0.27	−0.21	1.00										
IW	0.17	0.12	0.15	−0.28	−0.31	−0.21	−0.24	−0.38	−0.42	−0.27	0.33	−0.29	−0.31	−0.26	−0.31	−0.41	−0.26	0.55	1.00									
QW	−0.34	−0.20	−0.23	0.36	0.41	0.19	0.35	0.58	0.57	0.45	−0.49	0.54	0.49	0.41	0.49	0.67	0.43	−0.16	−0.33	1.00								
JS	−0.16	−0.14	−0.13	0.48	0.63	0.37	0.45	0.57	0.63	0.38	−0.43	0.55	0.49	0.43	0.48	0.73	0.56	−0.30	−0.41	0.59	1.00							
WF	0.49	0.42	0.36	−0.21	−0.15	−0.05	−0.14	−0.37	−0.39	−0.27	0.45	−0.32	−0.34	−0.32	−0.34	−0.48	−0.22	0.17	0.36	−0.43	−0.38	1.00						
TE	−0.13	−0.13	−0.15	0.25	0.27	0.17	0.23	0.32	0.41	0.24	−0.28	0.34	0.33	0.45	0.51	0.41	0.22	−0.20	−0.25	0.42	0.35	−0.26	1.00					
TM	−0.18	−0.15	−0.15	0.38	0.43	0.24	0.35	0.61	0.65	0.40	−0.44	0.60	0.57	0.46	0.52	0.64	0.40	−0.24	−0.39	0.61	0.58	−0.35	0.53	1.00				
JU	−0.22	−0.18	−0.18	0.45	0.46	0.23	0.32	0.65	0.74	0.38	−0.42	0.71	0.60	0.45	0.51	0.68	0.41	−0.18	−0.36	0.64	0.62	−0.38	0.48	0.76	1.00			
GH	−0.13	−0.11	−0.15	0.22	0.25	0.15	0.17	0.26	0.29	0.17	−0.20	0.23	0.21	0.23	0.29	0.32	0.30	−0.18	−0.25	0.27	0.35	−0.35	0.23	0.24	0.27	1.00		
ST	0.40	0.34	0.33	−0.22	−0.18	−0.10	−0.16	−0.35	−0.38	−0.26	0.43	−0.32	−0.31	−0.31	−0.35	−0.48	−0.27	0.17	0.32	−0.41	−0.38	0.66	−0.26	−0.33	−0.37	−0.41	1.00	
BO	0.34	0.30	0.34	−0.29	−0.26	−0.17	−0.20	−0.39	−0.42	−0.25	0.44	−0.34	−0.33	−0.31	−0.35	−0.51	−0.34	0.21	0.37	−0.42	−0.46	0.66	−0.26	−0.35	−0.40	−0.50	0.79	1.00

≥0.04 are statistically significant, *p* < 0.05; ≥0.05 are statistically significant, *p* < 0.01; ≥0.06 are statistically significant, *p* < 0.001. ^1^ Abbreviations of dimensions explained in Table 2.

**Table 6:** Pearson bivariate intercorrelations between scales in the Swedish Standard version of COPSOQ III based on data from 51 organizations. Correlations at the organizational level are presented in the lower left part of the table and correlations at the individual level in the upper right part.

Dimension ^1^	QD	WP	ED	IN	PD	VA	MW	PR	RE	CL	CO	QL	SS	SC	SW	CW	WE	JI	IW	QW	JS	WF	TE	TM	JU	GH	ST	BO
QD		0.48	0.37	−0.12	−0.10	0.01	−0.09	−0.28	−0.21	−0.27	0.40	−0.23	−0.23	−0.19	−0.18	−0.28	−0.12	0.01	0.21	−0.31	−0.28	0.43	−0.10	−0.22	−0.27	−0.24	0.41	0.39
WP	0.53		0.34	−0.21	−0.12	−0.06	−0.09	−0.26	−0.23	−0.11	0.38	−0.20	−0.21	−0.15	−0.12	−0.29	−0.01	−0.03	0.17	−0.22	−0.25	0.38	−0.09	−0.25	−0.27	−0.13	0.37	0.34
ED	0.46	0.30		−0.04	−0.01	0.09	0.02	−0.20	−0.16	−0.15	0.34	−0.19	−0.16	−0.14	−0.16	−0.23	−0.01	−0.05	0.19	−0.20	−0.20	0.37	−0.13	−0.20	−0.24	−0.22	0.37	0.37
IN	0.08	−0.37	0.04		0.60	0.49	0.45	0.46	0.54	0.23	−0.33	0.48	0.44	0.40	0.39	0.54	0.38	−0.22	−0.34	0.34	0.53	−0.27	0.30	0.48	0.53	0.26	−0.29	−0.32
PD	0.31	−0.11	0.25	0.78		0.55	0.61	0.50	0.58	0.30	−0.35	0.52	0.46	0.44	0.44	0.60	0.53	−0.26	−0.35	0.42	0.68	−0.28	0.38	0.52	0.54	0.28	−0.33	−0.34
VA	0.37	−0.19	0.39	0.70	0.73		0.48	0.24	0.35	0.11	−0.21	0.29	0.27	0.27	0.30	0.39	0.40	−0.20	−0.20	0.26	0.41	−0.11	0.26	0.32	0.31	0.19	−0.21	−0.23
MW	0.15	−0.21	0.49	0.64	0.74	0.75		0.38	0.46	0.37	−0.35	0.38	0.35	0.37	0.36	0.49	0.59	−0.24	−0.24	0.39	0.55	−0.24	0.29	0.41	0.40	0.26	−0.30	−0.31
PR	−0.17	−0.42	−0.22	0.56	0.49	0.22	0.40		0.71	0.48	−0.51	0.66	0.59	0.37	0.40	0.65	0.35	−0.17	−0.41	0.52	0.62	−0.38	0.32	0.69	0.70	0.27	−0.40	−0.40
RE	−0.08	−0.35	−0.03	0.79	0.69	0.48	0.60	0.80		0.41	−0.45	0.67	0.65	0.48	0.48	0.72	0.44	−0.26	−0.44	0.51	0.67	−0.37	0.41	0.73	0.78	0.33	−0.41	−0.42
CL	−0.40	−0.06	0.04	−0.06	−0.12	−0.17	0.14	0.43	0.23		−0.35	0.41	0.37	0.31	0.34	0.38	0.31	−0.16	−0.31	0.44	0.43	−0.30	0.19	0.37	0.41	0.20	−0.30	−0.27
CO	0.39	0.57	0.35	−0.54	−0.44	−0.27	−0.38	−0.75	−0.64	−0.23		−0.45	−0.42	−0.29	−0.31	−0.52	−0.34	0.12	0.35	−0.44	−0.52	0.46	−0.25	−0.49	−0.49	−0.27	0.44	0.43
QL	−0.12	−0.33	−0.10	0.58	0.60	0.29	0.38	0.79	0.72	0.23	−0.64		0.79	0.41	0.39	0.63	0.36	−0.13	−0.38	0.48	0.60	−0.30	0.35	0.65	0.71	0.28	−0.35	−0.37
SS	−0.19	−0.38	−0.08	0.55	0.54	0.24	0.48	0.86	0.76	0.37	−0.72	0.86		0.45	0.41	0.59	0.35	−0.13	−0.37	0.45	0.57	−0.31	0.33	0.63	0.66	0.28	−0.35	−0.36
SC	0.13	−0.13	0.07	0.68	0.55	0.46	0.45	0.40	0.60	0.10	−0.29	0.43	0.45		0.66	0.46	0.36	−0.20	−0.27	0.39	0.45	−0.30	0.50	0.38	0.46	0.27	−0.32	−0.29
SW	0.01	−0.18	−0.07	0.60	0.44	0.48	0.33	0.30	0.49	−0.06	−0.26	0.42	0.37	0.75		0.51	0.36	−0.25	−0.28	0.44	0.48	−0.26	0.52	0.40	0.47	0.29	−0.36	−0.33
CW	−0.09	−0.36	−0.13	0.75	0.68	0.52	0.51	0.82	0.85	0.26	−0.69	0.81	0.77	0.58	0.59		0.50	−0.21	−0.41	0.54	0.76	−0.43	0.40	0.69	0.71	0.34	−0.49	−0.51
WE	0.10	−0.11	0.36	0.52	0.61	0.64	0.83	0.30	0.49	0.18	−0.33	0.35	0.42	0.49	0.39	0.46		−0.11	−0.21	0.34	0.55	−0.32	0.24	0.39	0.38	0.34	−0.37	−0.41
JI	−0.53	−0.44	−0.34	−0.38	−0.48	−0.48	−0.38	−0.01	−0.31	0.03	−0.20	0.04	0.08	−0.43	−0.34	−0.24	−0.31		0.55	−0.14	−0.23	0.16	−0.23	−0.22	−0.19	−0.25	0.20	0.22
IW	0.01	0.01	0.29	−0.52	−0.42	−0.21	−0.17	−0.49	−0.56	−0.26	0.41	−0.33	−0.33	−0.46	−0.36	−0.61	−0.10	0.56		−0.32	−0.44	0.31	−0.26	−0.46	−0.43	−0.27	0.39	0.38
QW	−0.17	−0.29	−0.08	0.64	0.54	0.42	0.49	0.73	0.79	0.44	−0.70	0.64	0.71	0.67	0.55	0.79	0.44	−0.28	−0.60		0.56	−0.35	0.38	0.55	0.55	0.31	−0.40	−0.38
JS	−0.13	−0.37	−0.11	0.64	0.64	0.49	0.55	0.81	0.74	0.30	−0.76	0.68	0.77	0.41	0.44	0.87	0.42	−0.14	−0.60	0.75		−0.48	0.39	0.65	0.66	0.41	−0.52	−0.53
WF	0.31	0.41	0.51	−0.42	−0.21	−0.03	−0.14	−0.50	−0.49	−0.03	0.59	−0.33	−0.55	−0.34	−0.32	−0.46	−0.24	0.00	0.44	−0.48	−0.65		−0.20	−0.37	−0.40	−0.43	0.66	0.63
TE	0.22	−0.12	0.00	0.65	0.59	0.52	0.35	0.26	0.53	−0.17	−0.24	0.34	0.31	0.72	0.78	0.58	0.23	−0.48	−0.46	0.55	0.45	−0.23		0.43	0.49	0.25	−0.27	−0.27
TM	−0.17	−0.37	−0.16	0.66	0.60	0.35	0.49	0.89	0.90	0.33	−0.74	0.76	0.83	0.44	0.36	0.85	0.35	−0.19	−0.60	0.75	0.85	−0.57	0.42		0.81	0.30	−0.40	−0.41
JU	−0.15	−0.36	−0.17	0.79	0.65	0.41	0.49	0.81	0.94	0.26	−0.69	0.79	0.78	0.66	0.54	0.90	0.44	−0.27	−0.62	0.84	0.76	−0.52	0.56	0.89		0.33	−0.43	−0.44
GH	−0.17	0.04	−0.29	0.24	0.19	0.11	0.21	0.32	0.38	0.23	−0.33	0.24	0.34	0.24	0.32	0.41	0.48	−0.25	−0.56	0.39	0.47	−0.54	0.17	0.45	0.39		−0.52	−0.57
ST	0.30	0.41	0.40	−0.36	−0.28	−0.24	−0.28	−0.56	−0.58	−0.10	0.62	−0.46	−0.57	−0.31	−0.48	−0.62	−0.08	−0.06	0.45	−0.55	−0.67	0.77	−0.36	−0.64	−0.58	−0.54		0.81
BO	0.21	0.39	0.41	−0.51	−0.40	−0.37	−0.30	−0.57	−0.63	−0.03	0.62	−0.56	−0.53	−0.29	−0.46	−0.72	−0.28	0.06	0.55	−0.50	−0.69	0.61	−0.37	−0.70	−0.64	−0.60	0.83	

Individual level correlations ≥ 0.05 are statistically significant, *p* < 0.05; ≥0.07 are statistically significant, *p* < 0.01; ≥0.10 are statistically significant, *p* < 0.001. Organizational level correlations ≥ 0.29 are statistically significant, *p* < 0.05; ≥0.38 are statistically significant, *p* < 0.01; ≥0.44 are statistically significant, *p* < 0.001. ^1^ Abbreviations of dimensions explained in Table 2.

**Table 7:** Intraclass correlation coefficients (ICC(1) and ICC(2) *) for aggregation to occupational major group and for aggregation to organizational level (51 workplace surveys). For the workplace sample scale score: Mean and standard deviation, minimum, maximum, range and difference between the mean in the Workplace Survey compared to the weighted mean in the National Survey.

Dimension ^1^	Aggregation to ISCO-Major Occupational Group		Aggregation to Workplace Level							
	National Survey		Workplace Surveys							
	ICC(1) ^2^	ICC(2) ^3^	ICC(1) ^2^	ICC(2) ^3^	Mean	SD	Min	Max	Range	Difference to Benchmark ^4^
QD	0.11	0.98	0.11	0.82	43.3	9.3	19.6	60.7	41.2	2.4
WP	0.00	0.62	0.13	0.85	58.6	9.0	37.5	78.1	40.6	−1.3
ED	0.15	0.98	0.28	0.93	45.7	14.0	22.0	80.2	58.2	−1.1
IN	0.09	0.97	0.12	0.82	47.0	7.3	30.6	60.9	30.3	−3.2
PD	0.11	0.98	0.12	0.83	63.1	9.1	39.5	78.1	38.6	−7.3
VA	0.11	0.98	0.16	0.88	67.9	11.5	31.6	85.2	53.6	−0.1
MW	0.07	0.96	0.14	0.85	78.6	8.8	52.4	90.6	38.3	0.3
PR	0.02	0.85	0.14	0.85	55.2	9.3	35.2	68.8	33.5	−5.0
RE	0.02	0.86	0.09	0.79	60.1	8.6	41.3	77.4	36.2	−5.5
CL	0.03	0.90	0.06	0.69	70.6	7.0	39.6	85.8	46.3	−7.5
CO	0.01	0.75	0.10	0.80	40.7	7.7	29.2	60.2	31.1	−1.5
QL	0.01	0.66	0.13	0.83	57.0	11.0	32.6	79.4	46.8	2.9
SS	0.00	0.51	0.10	0.80	76.8	9.9	57.4	93.8	36.4	1.5
SC	0.01	0.76	0.05	0.63	77.4	7.2	56.3	89.2	33.0	−2.8
SW	0.01	0.67	0.07	0.73	77.5	6.7	56.7	88.6	32.0	−2.4
CW	0.01	0.82	0.17	0.86	61.0	12.4	33.9	87.5	53.6	−3.7
WE	0.03	0.90	0.05	0.63	70.7	6.5	58.3	81.7	23.3	1.3
JI	0.08	0.97	0.12	0.82	19.9	8.5	3.8	40.1	36.3	−0.3
IW	0.01	0.82	0.06	0.70	25.5	8.3	10.6	45.0	34.4	0.6
QW	0.00	0.56	0.09	0.78	66.7	9.8	37.5	87.5	50.0	−1.5
JS	0.03	0.91	0.10	0.81	64.6	7.4	43.3	77.6	34.4	0.2
WF	0.02	0.90	0.06	0.69	36.1	9.0	17.6	50.6	33.0	−3.6
TE	0.02	0.86	0.15	0.86	68.1	10.5	37.5	87.5	50.0	−3.2
TM	0.02	0.85	0.25	0.92	66.6	11.3	43.1	88.1	45.0	−2.7
JU	0.02	0.88	0.19	0.89	56.0	10.7	32.5	75.2	42.7	−3.7
GH	0.02	0.87	0.02	0.39	57.1	5.9	45.2	68.0	22.8	−4.2
ST	0.01	0.74	0.05	0.65	33.0	7.7	15.8	51.5	35.7	−3.0
BO	0.02	0.86	0.05	0.66	34.6		16.7	49.0	32.3	−1.6

^1^ Abbreviations of dimensions explained in Table 2. ^2^ ICC(1) represents the amount of variance in the employees’ responses that can be explained by their membership of a group (occupational/workplace. ^3^ ICC(2) is an estimate of reliability of the aggregated group means. ^4^ Benchmark presented in Table 2.

## Supplementary Materials

The following are available online at https://www.mdpi.com/1660-4601/17/9/3179/s1, Changes in items and dimensions from the Swedish version of COPSOQ II to COPSOQ III.

## Appendix A

**Table A1:** Characteristics of the study population based on ISCO-08 Major occupational groups (random sample).

ISCO-08 Major Occupational Groups	N	Women	Private Sector	Public Sector	Fixed Employment	Age (Years)		Income Level (Swedish Kronor Per Year before Income Tax)		Relational Work	Non-Managerial Position
		%	%	%	%	Mean	SD	Mean	SD	%	%
0. Armed Forces Occupations	5										
1. Managers	222	45.5	53.9	40.1	98.6	50.0	8.3	677,430	315,167	81.0	11.7
2. Professionals	987	65.2	33.0	62.9	95.8	47.1	10.7	457,505	191,216	84.6	67.7
3. Technicians and Associate Professionals	421	43.5	60.7	32.2	98.1	46.6	10.6	442,773	160,008	83.0	69.8
4. Clerical Support Workers	220	70.9	56.9	33.5	94.0	47.7	10.5	334,546	104,186	70.8	78.5
5. Services and Support Workers	482	77.2	34.6	61.1	86.9	48.7	11.8	292,795	89,345	96.0	77.8
6. Skilled Agricultural, Forestry and Fishery Workers	20	45.0	55.0	30.0	75.0	47.7	12.5	265,315	82,340	45.0	75.0
7. Craft and Related Trades Workers	168	7.7	86.6	11.0	97.0	47.1	10.7	387,394	102,623	72.7	75.6
8. Plant and Machine Operators and Assemblers	152	17.8	86.3	8.2	93.8	49.8	10.4	373,195	111,118	50.3	78.9
9. Elementary Occupations	88	64.8	44.0	42.9	87.4	49.3	11.0	262,938	77,095	78.4	77.3
Not classified	82										

## Appendix B

**Table A2:** Overview of dimensions included in the Swedish standard version of COPSOQ III and its correspondence with the International COPSOQ III and with the existing Swedish middle version of COPSOQ II.

The Swedish Standard COPSOQ III.	No. of Items	Correspondence with the International COPSOQ III	Correspondence with the Existing Swedish COPSOQ II
Quantitative demands	3	Equal to MIDDLE	1 item shorter
Work pace	2	Equal to MIDDLE	1 item shorter
Emotional demands	3	Equal to MIDDLE	1 item shorter, 1 item changed wording
Influence	4	1 MIDDLE item replaced by 1 LONG item	No changes
Possibilities for development	3	Equal to MIDDLE	1 item shorter
Variation of work	1	1 out of 2 LONG items	No changes
Meaning of work	1	Equal to CORE	2 items shorter
Predictability	2	Equal to MIDDLE	No changes
Recognition	2	1 CORE item supplemented by 1 LONG item	1 item shorter, dimension name changed
Role clarity	3	Equal to MIDDLE	Dimension name changed
Role conflicts	3	2 CORE items supplemented by 1 MIDDLE item on illegitimate tasks	1 item shorter
Quality of leadership	3	Equal to MIDDLE	1 item shorter, dimension name changed
Social support from supervisor	2	Equal to MIDDLE	1 item shorter
Social support from colleagues	2	Equal to MIDDLE	1 item shorter
Sense of community at work	3	Equal to LONG	No changes
Commitment to the workplace	3	3 out of 5 LONG items	1 item replaced; dimension name changed
Work engagement	3	Equal to LONG	New dimension
Job insecurity	3	Equal to LONG	New dimension
Insecurity over working conditions	2	1 CORE item supplemented by 1 LONG item	New dimension
Quality of work	2	Equal to LONG	New dimension
Job satisfaction	4	4 out of 5 LONG items (1 MIDDLE item excluded)	No changes
Work life conflict	3	2 CORE items supplemented by 1 LONG item	1 item shorter, 1 item replaced, response options changed
Horizontal trust	1	Equal to MIDDLE	2 items shorter, dimension name changed
Vertical trust	3	Equal to MIDDLE	1 item shorter, 2 items changed wording, dimension name changed
Organizational justice	3	2 CORE items supplemented by 1 LONG item	1 item shorter, 2 items changed wording, dimension name changed
Self-rated health	1	Equal to CORE	No changes
Stress	3	Equal to LONG	1 item shorter
Burnout SE	3	3 out of 4 LONG version items	1 item shorter
Threats of violence	1 (2)	Equal to LONG	No changes
Physical violence	1 (2)	Equal to LONG	No changes
Bullying	1 (2)	Equal to LONG	No changes
Sexual harassment	1 (2)	Equal to LONG	No changes
Cyber Bullying	1 (2)	Equal to LONG	New dimension
