# Supplementary material for: Validation of the Copenhagen Psychosocial Questionnaire Version III and Establishment of Benchmarks for Psychosocial Risk Management in Sweden
Source: Int J Environ Res Public Health. 2020 May 2;17(9):3179. doi: 10.3390/ijerph17093179 (PMC7246423; doi:10.3390/ijerph17093179)
Supplement: Supplementary file 1 [file ijerph-17-03179-s001.pdf]

Supplementary Material. Changes in items and dimensions from the Swedish version of COPSOQ II to COPSOQ III

Dimension English	Dimension Swedish	Item name	Level interna-tional COPSOQ III	Tested in cognitive interviews	Included in SCB survey	Swedish standard COPSOQ III	Item changes from Swedish COPSOQ II to COPSOQ III	Item in English	Item in Swedish	Response options*
**Demands at Work**										
Quantitative Demands	Kvantitativa krav	QD1	MIDDLE	x	x	x		Is your workload unevenly distributed so it piles up?	Är din arbetsbörda ojämnt fördelad så att arbete samlas på hög?	1
		QD2	CORE	x	x	x		How often do you not have time to complete all your work tasks?	Hur ofta händer det att du inte hinner slutföra alla dina arbetsuppgifter?	1
		QD3	CORE	x	x	x		Do you get behind with your work?	Kommer du efter med ditt arbete?	1
		QD4	LONG	x			QD4 was included in MIDDLE COPSOQ II, now only in LONG version	Do you have enough time for your work tasks?	Har du tillräckligt med tid för att utföra dina arbetsuppgifter?	1R
Work Pace	Arbetstempo	WP1	CORE	x	x	x		Do you have to work very fast?	Är du tvungen att arbeta väldigt snabbt?	1
		WP2	CORE	x	x	x		Do you work at a high pace throughout the day?	Arbetar du i ett högt tempo under hela dagen?	2
		WP3	LONG	x			WP3 was included in MIDDLE COPSOQ II, now only in LONG version	Is it necessary to keep working at a high pace?	Måste du hålla ett högt arbetstempo?	2
Cognitive Demands	Kognitiva krav	CD1	LONG	**			Translation by Wentz et al. 2019**	Do you have to keep your eyes on lots of things while you work?	Måste du hålla reda på manga saker samtidigt I ditt arbete?	1
		CD2	LONG	**			Translation by Wentz et al. 2019**	Does your work require that you remember at lot of things?	Kräver ditt arbete att du ska komma ihåg mycket?	1
		CD3	LONG	**			Translation by Wentz et al. 2019**	Does your work demand that you are good at coming up with new ideas?	Kräver ditt arbete att du är bra på att komma på nya idéer?	1
		CD4	LONG	**			Translation by Wentz et al. 2019**	Does your work require you to make difficult decisions?	Kräver ditt arbete att du fattar svåra beslut?	1
Emotional Demands	Känslomässiga krav	ED1	MIDDLE	x	x	x		Does your work put you in emotionally disturbing situations?	Hamnar du genom ditt arbete i känslomässigt påfrestande situationer?	1
		EDX2	CORE	x	x	x	"förhålla dig till" changed to "hantera" to match the change internationally	Do you have to deal with other people's personal problems as part of your work?	Måste du hantera andra människors personliga problem i ditt arbete?	1

		ED3	CORE	x	x	x		Is your work emotionally demanding?	Är ditt arbete känslomässigt krävande?	2
							ED4 not included in COPSOQ III			
Demands for Hiding Emotions	Krav om att dölja känslor	HE1	LONG					Are you required to treat everyone equally, even if you do not feel like it?		1
		HE2	MIDDLE	x				Does your work require that you hide your feelings?	Måste du dölja dina känslor i ditt arbete?	2
		HE3	MIDDLE	x				Are you required to be kind and open towards everyone – regardless of how they behave towards you?	Förväntas du vara vänlig och tillmötesgående mot alla, oavsett hur de beter sig mot dig?	2
		HE4	MIDDLE					Does your work require that you do not state your opinion?		1
**Work Organization and Job Contents**										
Influence at Work	Inflytande	INX1	CORE	x	x	x	International COPSOQ III congruent with the Swedish COPSOQ II, no changes made	Do you have a large degree of influence on the decisions concerning your work?	Har du möjlighet att påverka väsentliga beslut som gäller ditt arbete?	1
		IN2	LONG	x	x	x		Do you have a say in choosing who you work with?	Kan du påverka vem du arbetar tillsammans med?	1
		IN3	MIDDLE	x	x	x		Can you influence the amount of work assigned to you?	Kan du påverka din arbetsmängd?	1
		IN4	MIDDLE	x	x	x		Do you have any influence on what you do at work?	Kan du påverka vad du gör i ditt arbete?	1
		IN5	LONG	x				Can you influence how quickly you work?	Kan du påverka hur snabbt du arbetar?	1
		IN6	MIDDLE					Do you have any influence on HOW you do your work?		1
Possibilities for Development	Utvecklings-möjligheter						PD1 not included in COPSOQ III			
		PD2	CORE	x	x	x		Do you have the possibility of learning new things through your work?	Har du möjlighet att lära dig något nytt genom ditt arbete?	2
		PD3	CORE	x	x	x		Can you use your skills or expertise in your work?	Kan du använda ditt kunnande eller dina färdigheter i ditt arbete?	2
		PD4	MIDDLE	x	x	x		Does your work give you the opportunity to develop your skills?	Erbjuder ditt arbete möjligheter att utveckla dina färdigheter?	2
Variation of Work	Variation i arbetet	VA1	LONG	x	x	x		Is your work varied?	Är ditt arbete varierat?	1
		VA2	LONG	x	x		New item in Swedish LONG COPSOQ III	Do you have to do the same thing over and over again?	Innebär ditt arbete att man gör samma sak om och om igen?	1R

Control over Working Time	Kontroll över arbetstider						Dimension reintroduced from COPSOQ I, not included in the Swedish COPSOQ III			
		CT1	MIDDLE				Item reintroduced from COPSOQ I, not included in the Swedish COPSOQ III	Can you decide when to take a break?		1
		CT2	MIDDLE				Item reintroduced from COPSOQ I, not included in the Swedish COPSOQ III	Can you take holidays more or less when you wish?		1
		CT3	MIDDLE				Item reintroduced from COPSOQ I, not included in the Swedish COPSOQ III	Can you leave your work to have a chat with a colleague?		1
		CT4	MIDDLE				Item reintroduced from COPSOQ I, not included in the Swedish COPSOQ III	If you have some private business is it possible for you to leave your piece of work for half an hour without special permission?		1
		CT5	LONG				Item reintroduced from COPSOQ I, not included in the Swedish COPSOQ III	Do you have to do overtime?		1R
Meaning of Work	Mening i arbetet	MW1	CORE	x	x	x	International COPSOQ III congruent with the Swedish COPSOQ II	Is your work meaningful?	Är ditt arbete meningsfullt?	2
		MW2	MIDDLE	x				Do you feel that the work you do is important?	Känner du att din arbetsinsats är viktig?	2
							MW3 not included in COPSOQ III			
**Interpersonal Relations and Leadership**										
Predictability	Förutsägbarhet	PR1	CORE	x	x	x		At your place of work, are you informed well in advance concerning for example important decisions, changes or plans for the future?	Får du information i god tid på din arbetsplats t.ex. när det gäller viktiga beslut, förändringar och framtidsplaner?	2
		PR2	CORE	x	x	x		Do you receive all the information you need in order to do your work well?	Får du veta allt du behöver för att klara ditt arbete på ett bra sätt?	2

Recognition	Erkännande						Dimension name changed in Swedish COPSOQ III from *Belöning* to *Erkännande* following the change internationally			
		RE1	CORE	x	x	x		Is your work recognized and appreciated by the management?	Uppmärksammar och uppskattar ledningen din arbetsinsats?	2
		RE2	LONG	x				Does the management at your workplace respect you?	Respekteras du av ledningen på din arbetsplats?	2
		RE3	LONG	x	x	x		Are you treated fairly at your workplace?	Behandlas du rättvist på din arbetsplats?	2
Role Clarity	Rolltydlighet						Dimension name changed in Swedish COPSOQ III from *Klarhet i rollerna* to *Rolltydlighet*			
		CL1	CORE	x	x	x		Does your work have clear objectives?	Finns det klara mål för ditt arbete?	2
		CL2	MIDDLE	x	x	x		Do you know exactly which areas are your responsibility?	Vet du exakt vilka som är dina ansvarsområden?	2
		CL3	MIDDLE	x	x	x		Do you know exactly what is expected of you at work?	Vet du precis vad som förväntas av dig i ditt arbete?	2
Role Conflicts	Rollkonflikter						Dimension includes IT1 in Swedish COPSOQ III.			
							CO1 not included in COPSOQ III			
		CO2	CORE	x	x	x		Are contradictory demands placed on you at work?	Ställs det krav som strider mot varandra i ditt arbete?	2
		CO3	CORE	x	x	x		Do you sometimes have to do things which ought to have been done in a different way?	Måste du ibland göra något som egentligen borde ha gjorts annorlunda?	2
		IT1	MIDDLE	x	x	x	Variable name changed from *CO4* to *IT1* in COPSOQ III	Do you sometimes have to do things which seem to be unnecessary?	Måste du ibland göra saker i ditt arbete som kan verka onödiga?	2
Quality of Leadership	Ledarskapskvalitet						Dimension name changed in Swedish COPSOQ III from "Ledningskvalitet" to "Ledarskapskvalitet"	To what extent would you say that your immediate superior ...	I vilken utsträckning anser du att din närmaste chef	
		QLX1	MIDDLE	x	x	x	International COPSOQ III congruent with the Swedish COPSOQ II	makes sure that the members of staff have good development opportunities?	ser till att samtliga medarbetare har bra utvecklingsmöjligheter?	2

		QL2	LONG	x	x			gives high priority to job satisfaction?	prioriterar trivseln på arbetsplatsen högt?	2
		QL3	CORE	x	x	x		is good at work planning?	är bra på att planera arbetet?	2
		QL4	CORE	x	x	x		is good at solving conflicts?	är bra på att hantera konflikter?	2
Social Support from Supervisor	Socialt stöd från överordnad	SSX1	MIDDLE	x	x	x	International COPSOQ III congruent with the Swedish COPSOQ II	How often is your immediate superior willing to listen to your problems at work, if needed?	Om du behöver är din närmaste chef beredd att lyssna på problem som rör ditt arbete?	1
		SSX2	CORE	x	x	x	International COPSOQ III congruent with the Swedish COPSOQ II	How often do you get help and support from your immediate superior, if needed?	Om du behöver, får du stöd och hjälp med ditt arbete från din närmaste chef?	1
		SSX3	LONG	x				How often does your immediate superior talk with you about how well you carry out your work?	Hur ofta talar din närmaste chef med dig om hur du utför ditt arbete?	1
Social Support from Colleagues	Socialt stöd från kollegor	SCX1	CORE	x	x	x	International COPSOQ III congruent with the Swedish COPSOQ II	How often do you get help and support from your colleagues, if needed?	Om du behöver, får du hjälp och stöd från dina kollegor?	1
		SCX2	MIDDLE	x	x	x	International COPSOQ III congruent with the Swedish COPSOQ II	How often are your colleagues willing to listen to your problems at work, if needed?	Om du behöver, är dina kollegor beredda att lyssna till dina problem med arbetet?	1
		SC3	LONG	x				How often do your colleagues talk with you about how well you carry out your work?		1
Sense of Community at Work	Social gemenskap i arbetet						Dimension name changed in International COPSOQ III, no changes made in the Swedish COPSOQ III			
		SW1	CORE	x	x	x		Is there a good atmosphere between you and your colleagues?	Är stämningen bra mellan dig och dina arbetskamrater?	1
		SW2	LONG	x	x	x		Is there good co-operation between the colleagues at work?	Är samarbetet bra mellan arbetskamraterna på din arbetsplats?	1
		SW3	MIDDLE	x	x	x		Do you feel part of a community at your place of work?	Känner du dig delaktig i en gemenskap på din arbetsplats?	1
**Work-Individual Interface**										
Commitment to the Workplace	Engagemang i organisationen						Dimension name changed in Swedish COPSOQ III from “Involvering på arbetsplatsen” to “Engagemang i organisationen”			

		CW1	LONG	x				Do you enjoy telling others about your place of work?		2
		CW2	LONG	x				Do you feel that your place of work is of great importance to you?	Tycker du att din arbetsplats har stor betydelse för dig?	2
		CWX3	LONG	x	x	x	International COPSOQ III congruent with the Swedish COPSOQ II	Would you recommend other people to apply for a position at your workplace?	Skulle du rekommendera andra att söka anställning på din arbetsplats?	2
		CW4	LONG	x	x	x		How often do you consider looking for work elsewhere?	Hur ofta överväger du att söka ett nytt jobb?	1R
		CW5	LONG	x	x	x	New item in Swedish STANDARD COPSOQ III	Are you proud of being part of this organization?	Är du stolt över att vara en del av den organisation som du arbetar inom?	2
Work Engagement	Arbetsengagemang	WE_T					New dimension in Swedish STANDARD COPSOQ III	How often do you experience the following?	Hur ofta upplever du följande?	
		WE1	LONG	x	x	x	New item in Swedish STANDARD COPSOQ III	At my work, I feel bursting with energy.	Jag känner mig full av energi när jag utför mitt arbete	3
		WE2	LONG	x	x	x	New item in Swedish STANDARD COPSOQ III	I am enthusiastic about my job.	Jag har ett brinnande intresse för mitt arbete	3
		WE3	LONG	x	x	x	New item in Swedish STANDARD COPSOQ III	I am immersed in my work.	Jag är starkt engagerad i mitt arbete	3
Job insecurity	Oro för arbetslöshet						New dimension in Swedish STANDARD COPSOQ III			
		JI1	CORE	x	x	x	New item in Swedish STANDARD COPSOQ III	Are you worried about becoming unemployed?	Oroar du dig för att bli arbetslös?	2
		JI2	LONG	x	x	x	New item in Swedish STANDARD COPSOQ III	Are you worried about new technology making you redundant?	Oroar du dig för att ny teknologi kan göra dig överflödig?	2
		JI3	CORE	x	x	x	New item in Swedish STANDARD COPSOQ III	Are you worried about it being difficult for you to find another job if you became unemployed?	Är du orolig för att det skulle bli svårt att hitta nytt arbete om du blev arbetslös?	2
Insecurity over Working Conditions	Oro för förändrade arbetsförhållanden						New dimension in Swedish STANDARD COPSOQ III			
		IW1	CORE	x	x	x	New item in Swedish STANDARD COPSOQ III	Are you worried about being transferred to another job against your will?	Är du orolig för att bli omplacerad mot din vilja?	2
		IW2	LONG	x	x	x	New item in Swedish STANDARD COPSOQ III	Are you worried about your working tasks being changed against your will?	Är du orolig för att dina arbetsuppgifter förändras mot din vilja?	2

		IW3	MIDDLE				New item in Swedish LONG COPSOQ III	Are you worried about the timetable being changed (shift, weekdays, time to enter and leave ...) against your will?		2
		IW4	MIDDLE				New item in Swedish LONG COPSOQ III	Are you worried about a decrease in your salary (reduction, variable pay being introduced ...)?		2
		IW5	LONG	x			New item in Swedish LONG COPSOQ III	Are there good prospects in your job?	Finns det goda framtidsutsikter i ditt jobb?	2
Quality of Work	Kvalitet i arbetet						New dimension in Swedish STANDARD COPSOQ III			
		QW1	LONG	x	x	x	New item in Swedish STANDARD COPSOQ III	To what extent do you find it possible to perform your work tasks at a satisfactory quality?	I vilken utsträckning anser du att det är möjligt att utföra dina arbetsuppgifter med en tillfredställande kvalitet?	2
		QW2	MIDDLE	x	x	x	New item in Swedish STANDARD COPSOQ III	Are you satisfied with the quality of the work performed at your workplace?	Är du nöjd med kvaliteten på det arbete som utförs på din arbetsplats?	2
Job Satisfaction	Tillfredsställelse med arbetet	JS_T						Regarding your work in general. How pleased are you with	Angående ditt arbete i allmänhet. Hur tillfredsställd är du med	
		JS1	MIDDLE	x	x	x		your work prospects?	dina framtidsutsikter i jobbet?	6
		JS2	LONG	x	x	x	Wording changed from *fysiska arbetsförhållandena* to *de fysiska arbetsförhållandena*	the physical working conditions?	de fysiska arbetsförhållandena?	6
		JS3	LONG	x	x	x		the way your abilities are used?	det sätt dina kunskaper används på?	6
		JS4	CORE	x	x	x		your job as a whole, everything taken into consideration?	ditt arbete som helhet, allt inräknat?	6
		JS5	MIDDLE	x			New item in Swedish LONG COPSOQ III	your salary?	din lön?	6
Work Life Conflict	Konflikt mellan arbete och privatliv	WF_T						The next four questions concern the ways in which your work affects your private life:	Följande frågor handlar om relationen mellan arbete och privatliv	
		WFX1	LONG	x			Response options changed	Are there times when you need to be at work and at home at the same time?	Händer det att du behöver vara på arbetet och hemma samtidigt?	1
		WF2	CORE	x	x	x	Response options changed	Do you feel that your work drains so much of your energy that it has a negative effect on your private life?	Känner du att ditt arbete tar så mycket av din energi att det påverkar privatlivet negativt?	2

		WF3	CORE	x	x	x	Response options changed	Do you feel that your work takes so much of your time that it has a negative effect on your private life?	Känner du att ditt arbete tar så mycket av din tid att det påverkar privatlivet negativt?	2
		WF5	LONG	x	x	x	New item in Swedish STANDARD COPSOQ III	The demands of my work interfere with my private and family life?	Känner du att kraven i ditt arbete stör privatlivet?	2
		WF6	LONG				Not included in the Swedish COPSOQ III	Due to work-related duties, I have to make changes to my plans for private and family activities.		2
**Social Capital**										
Horizontal Trust	Horisontell tillit	TE-T					Dimension name changed from *Tillit och trovärdighet mellan de anställda inbördes* to *Horisontell tillit,* following the international change	The next questions are not about your own job but about the workplace as a whole	Arbetsplatsen sett som en helhet. Frågorna nedan avser inte ditt eget jobb utan din arbetsplats som helhet	
		TE1	LONG	x	x		*De anställda* changed to *medarbetarna* in Swedish COPSOQ III	Do the employees withhold information from each other?	Undanhåller medarbetarna information från varandra?	2R
		TE2	LONG	x	x		*De anställda* changed to *medarbetarna* in Swedish COPSOQ III	Do the employees withhold information from the management?	Undanhåller medarbetarna information från ledningen?	2R
		TE3	MIDDLE	x	x	x	*De anställda* changed to *medarbetarna* in Swedish COPSOQ III	Do the employees in general trust each other?	Litar medarbetarna i allmänhet på varandra?	2
Vertical Trust	Vertikal tillit	TM_T					Dimension name changed from *Tillit och trovärdighet mellan ledning och medarbetare* to *Vertikal tillit,* following the international change	The next questions are not about your own job but about the workplace as a whole	Arbetsplatsen sett som en helhet. Frågorna nedan avser inte ditt eget jobb utan din arbetsplats som helhet	
		TM1	CORE	x	x	x		Does the management trust the employees to do their work well?	Litar ledningen på att medarbetarna gör ett bra jobb?	2
		TMX2	CORE	x	x	x	*Man* changed to *medarbetarna in Swedish COPSOQ III,* following the international change	Can the employees trust the information that comes from the management?	Kan medarbetarna lita på den information som kommer från ledningen?	2

		TM3	LONG	x	x		*De anställda* changed to *medarbetarna* in Swedish COPSOQ III	Does the management withhold important information from the employees?	Undanhåller ledningen viktig information från medarbetarna?	2R
		TM4	MIDDLE	x	x	x	*De anställda* changed to *medarbetarna* in Swedish COPSOQ III	Are the employees able to express their views and feelings?	Är det möjligt för medarbetarna att uttrycka sina åsikter och känslor?	2
Organizational Justice	Organisatorisk rättvisa	JU_T					Dimension name changed in Swedish COPSOQ III from *Rättvisa och respekt* to *Organisatorisk rättvisa,* following the international change	The next questions are not about your own job but about the workplace as a whole	Arbetsplatsen sett som en helhet. Frågorna nedan avser inte ditt eget jobb utan din arbetsplats som helhet	
		JU1	CORE	x	x	x	*Man* changed to *medarbetarna* in Swedish COPSOQ III, following the international change	Are conflicts resolved in a fair way?	Löses konflikter på ett rättvist sätt?	2
		JU2	LONG	x	x	x	*De anställda* changed to *medarbetarna* in Swedish COPSOQ III	Are employees appreciated when they have done a good job?	Blir medarbetarna uppskattade för en bra arbetsinsats?	2
		JU3	LONG	x			*De anställda* changed to *medarbetarna* in Swedish COPSOQ III	Are all suggestions from employees treated seriously by the management?	Hanterar ledningen alla förslag från de medarbetarna seriöst?	2
		JU4	CORE	x	x	x		Is the work distributed fairly?	Fördelas arbetsuppgifterna på ett rättvist sätt?	2
**Conflicts and offensive behaviors**										
Gossip and Slander	Skvaller och förtal	GS1	LONG	x			New item in Swedish LONG COPSOQ III	Have you been exposed to gossip and slander at your workplace during the last 12 months?	Har du under de senaste 12 månaderna varit utsatt för skvaller och förtal på din arbetsplats?	4
		GS2	LONG	x			New item in Swedish LONG COPSOQ III	If yes, from whom? (You may tick off more than one)	Om ja, från vem?	5M
Conflicts and Quarrels	Bråk eller konflikter	CQ1	LONG	x			New item in Swedish LONG COPSOQ III	Have you been involved in quarrels or conflicts at your workplace during the last 12 months?	Har du under de senaste 12 månaderna varit inblandad i bråk eller konflikter på din arbetsplats?	4
Unpleasant Teasing	Retad på jobbet	UT1	LONG	x			New item in Swedish LONG COPSOQ III	Have you been exposed to unpleasant teasing at your	Har du under de senaste 12 månaderna blivit retad på din arbetsplats?	4

								workplace during the last 12 months?		
		UT2	LONG	x			New item in Swedish LONG COPSOQ III	If yes, from whom? (You may tick off more than one)	Om ja, från vem?	5M
Cyber Bullying	Digitala kränkningar	HSM1	LONG	x	x	x	New item in Swedish STANDARD COPSOQ III	Have you been exposed to work-related harassment on the social media (e.g. Facebook), by e-mail or text messages during the last 12 months?	Har du under de senaste 12 månaderna blivit utsatt för kränkningar på sociala medier (t.ex. Facebook), via e-post eller SMS som har något med ditt arbete att göra?	4
		HSM2	LONG	x	x	x	New item in Swedish STANDARD COPSOQ III	If yes, from whom? (You may tick off more than one)	Om ja, från vem?	5M
Sexual Harassment	Icke önskvärd sexuell uppmärksamhet	SH1	LONG	x	x	x		Have you been exposed to undesired sexual attention at your workplace during the last 12 months?	Har du under de senaste 12 månaderna blivit utsatt för icke önskvärd sexuell uppmärksamhet på din arbetsplats?	4
		SH2	LONG	x	x	x		If yes, from whom? (You may tick off more than one)	Om ja, från vem?	5M
Threats of Violence	Hot om våld	TV1	LONG	x	x	x		Have you been exposed to threats of violence at your workplace during the last 12 months?	Har du under de senaste 12 månaderna blivit utsatt för hot om våld på din arbetsplats?	4
		TV2	LONG	x	x	x		If yes, from whom? (You may tick off more than one)	Om ja, från vem?	5M
Physical Violence	Fysiskt våld	PV1	LONG	x	x	x		Have you been exposed to physical violence at your workplace during the last 12 months?	Har du under de senaste 12 månaderna blivit utsatt för fysiskt våld på din arbetsplats?	4
		PV2	LONG	x	x	x		If yes, from whom? (You may tick off more than one)	Om ja, från vem?	5M
Bullying	Mobbning	TE_BU			x	x		Bullying means that a person repeatedly is exposed to unpleasant or degrading treatment, and that the person finds it difficult to defend himself or herself against it.	Med mobbing menas att man upprepade gånger blir utsatt för obehagliga eller kränkande handlingar, som det är svårt att försvara sig emot.	
		BU1	LONG	x	x	x		Have you been exposed to bullying at your workplace during the last 12 months?	Har du under de senaste 12 månaderna blivit utsatt för mobbing på din arbetsplats?	4
		BU3	LONG	x	x	x	Item name changed from BU2 to BU3 in Swedish COPSOQ III, following the international change	If yes, from whom? (You may tick off more than one)	Om ja, från vem?	5M
		BU2	LONG	x			Not included in the Swedish COPSOQ III	How often do you feel unjustly criticized, bullied or shown up in front of others by your colleagues or your superior?		1

Health and well-being

Self-Rated Health	Självskattad hälsa	GH1	CORE	x	x	x		In general, would you say your health is:	I allmänhet, skulle du vilja säga att din hälsa är:	7
		GH2	LONG	x				If you evaluate the best conceivable state of health at 10 points and the worst at 0 points: How many points do you then give your present state of health?		8
Sleeping Troubles	Sömnbesvär						Dimension was included in MIDDLE COPSOQ II, now only in LONG version	Top page: These questions are about how you have been during the last 4 weeks.	Följande frågor avser hur du har haft det under de senaste 4 veckorna.	
		SL1	LONG	x			SL1 was included in MIDDLE COPSOQ II, now only in LONG version	How often have you slept badly and restlessly?	Hur ofta har du sovit dåligt eller oroligt?	9
		SL2	LONG	x			SL2 was included in MIDDLE COPSOQ II, now only in LONG version	How often have you found it hard to go to sleep?	Hur ofta har du haft svårt att somna?	9
		SL3	LONG	x			SL3 was included in MIDDLE COPSOQ II, now only in LONG version	How often have you woken up too early and not been able to get back to sleep?	Hur ofta har du vaknat för tidigt och inte kunnat somna om?	9
		SL4	LONG	x			SL4 was included in MIDDLE COPSOQ II, now only in LONG version	How often have you woken up several times and found it difficult to get back to sleep?	Hur ofta har du vaknat flera gånger och haft svårt att somna om?	9
Burnout	Utbrändhet	BO_T	LONG	x	x	x		Top page: These questions are about how you have been during the last 4 weeks.	Följande frågor avser hur du har haft det under de senaste 4 veckorna.	
		BO1	LONG	x	x	x		How often have you felt worn out?	Hur ofta har du saknat ork och energi?	9
		BO2	LONG	x	x	x		How often have you been physically exhausted?	Hur ofta har du varit fysiskt utmattad?	9
		BO3	LONG	x	x	x		How often have you been emotionally exhausted?	Hur ofta har du varit känslomässigt utmattad?	9
		BO4	LONG	x			BO4 was included in MIDDLE COPSOQ II, now only in LONG version)	How often have you felt tired?	Hur ofta har du känt dig trött?	9

Stress	Stress	ST_T						Top page: These questions are about how you have been during the last 4 weeks.	Följande frågor avser hur du har haft det under de senaste 4 veckorna.	
		ST1	LONG	x	x	x		How often have you had problems relaxing?	Hur ofta har du haft svårt att koppla av?	9
		ST2	LONG	x	x	x		How often have you been irritable?	Hur ofta har du varit lättretlig?	9
		ST3	LONG	x	x	x		How often have you been tense?	Hur ofta har du varit anspänd?	9
							ST4 excluded from COPSOQ III		Hur ofta har du varit stressad?	
Somatic Stress	Somatisk stress	SO_T					New dimension in Swedish LONG COPSOQ III	Top page: These questions are about how you have been during the last 4 weeks.	Följande frågor avser hur du har haft det under de senaste 4 veckorna.	
		SO1	LONG	x			New item in Swedish LONG COPSOQ III	How often have you had stomach ache?	Hur ofta har du haft ont i magen?	9
		SO2	LONG	x			New item in Swedish LONG COPSOQ III	How often have you had a headache?	Hur ofta har du haft huvudvärk?	9
		SO3	LONG	x			New item in Swedish LONG COPSOQ III	How often have you had palpitations?	Hur ofta har du haft hjärtklappning?	9
		SO4	LONG				Not included in the Swedish COPSOQ III	How often have you had tension in various muscles?		9
Cognitive Stress	Kognitiv stress	CS_T					New dimension in Swedish LONG COPSOQ III	Top page: These questions are about how you have been during the last 4 weeks.	Följande frågor avser hur du har haft det under de senaste 4 veckorna.	
		CS1	LONG	**			Translation by Wentz et al. 2019**	How often have you had problems concentrating?	Hur ofta har du haft svårt att koncentrera dig?	9
		CS2	LONG	**			Translation by Wentz et al. 2019**	How often have you found it difficult to think clearly?	Hur ofta har du haft svårt att tänka klart?	9
		CS3	LONG	**			Translation by Wentz et al. 2019**	How often have you had difficulty in taking decisions?	Hur ofta har du haft svårt att fatta beslut?	9
		CS4	LONG	**			Translation by Wentz et al. 2019**	How often have you had difficulty with remembering?	Hur ofta har du haft svårt att minnas?	9
Depressive Symptoms	Depressiva symptom	DS_T	LONG	**			New dimension in Swedish LONG COPSOQ III	These questions are about how you have been during the last 4 weeks.		
		DS1	LONG	**			Translation by Wentz et al. 2019**	How often have you felt sad?	Hur ofta har du känt dig ledsen/nedstämd?	9
		DS2	LONG	**			Translation by Wentz et al. 2019**	How often have you lacked self-confidence?	Hur ofta har du saknat självförtroende?	9
		DS3	LONG	**			Translation by Wentz et al. 2019**	How often have you had a bad conscience or felt guilty?	Hur ofta har du haft dåligt samvete eller skuldkänslor?	9
		DS4	LONG	**			Translation by Wentz et al. 2019**	How often have you lacked interest in everyday things?	Hur ofta har du saknat intresse för sådant du gör till vardags?	9

*** Response options in English and in Swedish**

1: Always (100); Often (75); Sometimes (50); Seldom (25); Never/hardly ever (0)

1R: Always (0); Often (25); Sometimes (50); Seldom (75); Never/hardly ever (100) (Reversed scoring)

2: To a very large extent (100); To a large extent (75); Somewhat (50); To a small extent (25); To a very small extent (0)

2R: To a very large extent (0); To a large extent (25); Somewhat (50); To a small extent (25); To a very small extent (100) (Reversed scoring)

3: Never (0), Seldom (25), Sometimes (50), Often (75), Always (100)

4: Yes, daily; Yes, weekly; Yes, monthly; Yes, a few times; No

5M: Colleagues, Manager/superior, Subordinates, Clients/customers/patients (Multiple response options)

6: Very satisfied (100), Satisfied (75), Neither/Nor (50), Unsatisfied (25), Very unsatisfied (0)

7: Excellent (100), Very good (75), Good (50), Fair (25), Poor (0)

8:0, 1, 2, 4, 5, 6, 7, 8, 9, 10

9: All the time (100); A large part of the time (75); Part of the time (50); A small part of the time (25); Not at all (0)

1: Alltid (100); Ofta (75); Ibland (50); Sällan (25); Aldrig/nästan aldrig (0)

1R: Alltid (0); Ofta (25); Ibland (50); Sällan (75); Aldrig/nästan aldrig (100) (omvänd poängsättning)

2: I mycket hög grad (0); I hög grad (25); Delvis (50); I liten grad (75); I mycket liten grad (100)

2R: I mycket hög grad (100); I hög grad (75); Delvis (50); I liten grad (25); I mycket liten grad (0) (omvänd poängsättning)

3: Aldrig (0); Sällan (25); Ibland (50); Ofta (75); Alltid (100)

4: Ja, dagligen; Ja, varje vecka; Ja, varje månad; Ja, några gånger; Nej

5M: Kollegor; En överordnad; Underställda; Klienter/kunder/patienter

6: Mycket tillfredsställd (100); Tillfredsställd (75); Varken eller (50); Otillfredsställd (25); Mycket otillfredsställd (0)

7: Utmärkt (100); Mycket god (75); God (50); Någorlunda (25); Dålig (0)

8: 0, 1, 2, 4, 5, 6, 7, 8, 9, 10

9: Hela tiden (100); En stor del av tiden (75); En del av tiden (50); En liten del av tiden (25); Inte alls (0)

****** For further details regarding the translation and validation of the dimensions *Cognitive Demands, Cognitive Stress, Depression* and for citation: Wentz K, Gyllensten K, Sluiter J K, Hagberg M. (2019). Need for recovery in relation to effort from work and health in four occupations. International Archives of Occupational and Environmental Health, 1-17.
